# Adult obesity and mid-life physical functioning in two British birth cohorts: investigating the mediating role of physical inactivity

**DOI:** 10.1093/ije/dyaa014

**Published:** 2020-03-06

**Authors:** Snehal M Pinto Pereira, Bianca L De Stavola, Nina T Rogers, Rebecca Hardy, Rachel Cooper, Chris Power

**Affiliations:** d1 UCL Research Department of Epidemiology & Public Health, London WC1E 7HB, UK; d2 MRC Unit for Lifelong Health and Ageing at UCL, London WC1E 7HB, UK; d3 Population, Policy and Practice Research and Teaching Department, UCL Great Ormond Street Institute of Child Health, London WC1N 1EH, UK; d4 CLOSER, Department of Social Science, UCL Institute of Education, London WC1H 0AL, UK; d5 Musculoskeletal Science and Sports Medicine Research Centre, Department of Sport and Exercise Sciences, Faculty of Science and Engineering, Manchester Metropolitan University, Manchester M15 6BH, UK

**Keywords:** Obesity, physical inactivity, life-course, physical functioning, ageing, birth cohort, epidemiology

## Abstract

**Background:**

Associations between obesity and physical inactivity are bi-directional. Both are associated with physical functioning (PF, ability to perform physical tasks of daily living) but whether obesity influences PF via inactivity is unknown. We investigated whether mid-adult obesity trajectories were associated with subsequent PF and mediated by inactivity.

**Methods:**

Body mass index (BMI; kg/m²) and inactivity were recorded at: 36, 43, 53 and 60–64 years in the 1946 Medical Research Council (MRC) National Survey of Health and Development (1946-NSHD; *n* = 2427), and at 33, 42 and 50 years in the 1958 National Child Development Study (1958-NCDS; *n* = 8674). Poor PF was defined as the lowest (gender and cohort-specific) 10% on the Short-form 36 Physical Component Summary subscale at 60–64 years (1946-NSHD) and 50 years (1958-NCDS). Estimated randomized-interventional-analogue natural direct (rNDE) and indirect (rNIE) effects of obesity trajectories on PF via inactivity are expressed as risk ratios [overall total effect (rTE) is rNDE multiplied by rNIE].

**Results:**

In both cohorts, most individuals (∼68%) were never obese in adulthood, 16–30% became obese and ≤11% were always obese. In 1946-NSHD, rTE of incident obesity at 43 years (vs never) on poor PF was 2.32 (1.13, 3.51); at 53 years it was 1.53 (0.91, 2.15). rNIEs via inactivity were 1.02 (0.97, 1.07) and 1.02 (0.99, 1.04), respectively. Estimated rTE of persistent obesity from 36 years was 2.91 (1.14, 4.69), with rNIE of 1.03 (0.96, 1.10). In 1958-NCDS, patterns of association were similar, albeit weaker.

**Conclusions:**

Longer duration of obesity was associated with increased risk of poor PF. Inactivity played a small mediating role. Findings reinforce the importance of preventing and delaying obesity onset to protect against poor PF.

Key Messages

- In the two oldest British birth cohorts, obesity from the mid-30s age group, was associated with subsequent poor physical functioning: more detrimental associations were observed among those who had been obese for longer.
- We simulate the potential impact on the risk of poor physical functioning of altering the prevalence of inactivity among individuals who are obese to be in line with inactivity prevalence among individuals who are non-obese. We found that the effect of obesity on poor physical functioning was only weakly mediated by inactivity, suggesting that the influence of obesity on later physical functioning acts mainly via alternative pathways.
- Given the increasing prevalence of obesity at young ages, findings suggest that a high proportion of the future adult population will be at risk of poor physical functioning even before reaching older age. This highlights the need for interventions to avert obesity to start at young ages and continue throughout life.

## Introduction

The global population is ageing.^1^ For example, in England, the number of people aged >65 years was 9.5 million in 2014, and projected to be 11.5 million in 2024.^2^ Such improvements in longevity would, ideally, be accompanied by increases in disability-free life-expectancy. However, a major obstacle to achieving this, is loss of physical functioning (PF), i.e. the ability to perform the physical tasks of daily living. Maintaining PF enables individuals to remain independent for longer, with positive consequences for them, their families and society.^3^ Factors associated with poor PF include adult obesity^4–7^ and unhealthy behaviours including physical inactivity.^8–10^ Obesity is of particular interest because it is currently highly prevalent,^11^ and given recent trends, increasing proportions of the population reaching older age will have accumulated greater exposure to obesity throughout their lives. This is relevant because the effect of obesity may depend on an individual’s lifetime trajectory, with some studies suggesting that longer duration of obesity confers greater risk of poor PF from mid-adulthood onwards.^4^^,^^5^^,^^7^ Concurrently, cumulative benefıts of physical activity across adulthood on PF have been shown.^8^^,^^10^ Although obesity prevention is essential, a further priority is to minimize the associated burden on important ageing outcomes including PF. In this context, potentially modifiable factors such as physical inactivity might be considered.

Whereas studies of adiposity [typically assessed by body-mass index (BMI)] have shown detrimental associations with PF,^4–6^^,^^12^^,^^13^ it is unclear whether inactivity attenuates the adiposity–PF association because this is not usually examined explicitly. Studies of inactivity that account for adiposity,^8–10^^,^^12^^,^^13^ often find detrimental associations between inactivity and poor PF. Considering adiposity and inactivity together, one study reported that both high BMI and low activity were associated with poor PF,^12^ whereas another found that only BMI predicted functional decline.^13^ Such inconsistent findings could be due to complexities in the relationship between adiposity and inactivity.^14^ Notably, inactivity and adiposity are likely to have a bi-directional association, with several studies^15^^,^^16^ including those using Mendelian randomization,^16^ suggesting that adiposity causally influences subsequent inactivity. Adiposity may influence PF irrespective of activity level,^17^ but it is helpful to understand whether inactivity is a main intermediary through which adiposity affects subsequent PF. The latter question concerns whether inactivity mediates the adiposity–PF association and to answer this, two related challenges need to be addressed. First, as mentioned above, associations between adiposity and inactivity may be bi-directional. Second, most studies of adiposity, (in)activity and PF have been limited to measures at a single time-point and, to our knowledge, not with repeat measures in a life-course context. These omissions are important given that both an individual’s adiposity and inactivity status can change with age.

Failure to consider interrelationships between adiposity and inactivity over the life-course limits the potential of research to address questions relevant to health policy on PF. One such question is: would the risk of poor PF decrease if an intervention resulted in obese individuals having similar activity prevalence in adulthood as non-obese individuals? Understanding the benefits of reducing inactivity among those who are obese to promote healthy ageing may be useful in guiding resource allocation decisions. At the population level, obesity reduction policies have not been successful,^18^ although several approaches to increase physical activity have been identified.^19^ Therefore, investigating whether inactivity mediates the influence of obesity on PF is important.

**Figure 1.**
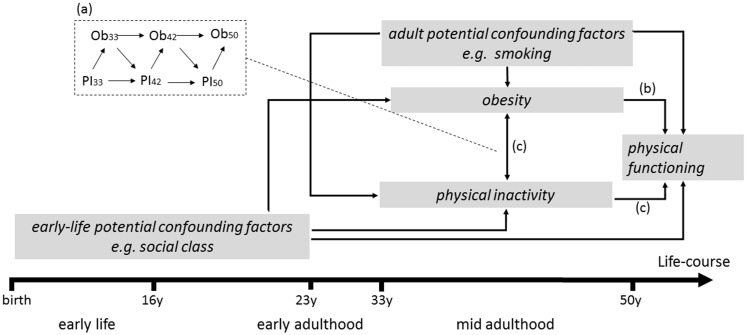
Simplified conceptual model representing a life-course pathway from mid-adulthood obesity and inactivity to physical functioning at 50 years. Ob, Obesity; PI, physical inactivity. Bi-directional associations between adult obesity and inactivity are represented in (**a**). In this study obesity is the exposure of interest and physical inactivity is the mediator of interest. Obesity trajectories in mid-adulthood could influence physical functioning either directly [as indicated by (**b**)] and/or indirectly, through obesity’s influence on subsequent inactivity [as indicated by (**c**)]. Potential confounding factors of the obesity–physical functioning and inactivity–physical functioning associations are represented as emanating from earlier life-stages (i.e. from early-life and early adulthood) as well as contemporaneously in mid-adulthood.

Using data from the two oldest British birth cohorts followed to late middle-age, with repeat and comparable measures of adult obesity and inactivity from their mid-30s onwards, we investigated associations between mid-adult obesity trajectories and subsequent PF, and whether associations were mediated by inactivity. By examining two birth cohorts we were able to assess whether findings were similar across generations. We hypothesized that longer duration of obesity from mid-adulthood would increase the risk of subsequent poor PF and that inactivity would mediate the association. Figure 1 illustrates our conceptual model, by showing the possible life-course pathways from obesity to mid-adult PF that involve physical inactivity. Although not explicit in Figure 1, obesity is the exposure of interest, with inactivity as a possible mediator. Bi-directional adult obesity and inactivity associations are acknowledged (Box 1a, Figure 1a), and the influence of mid-adult obesity trajectories on PF is shown as direct (via 1b, Figure 1b) and/or indirect, through obesity’s influence on subsequent inactivity (1c, Figure 1c). In addition, potential confounding factors are represented as emanating from earlier life-stages as well as contemporaneously in adulthood.

## Methods

The two British birth cohort studies used here, initiated in 1946 [Medical Research Council (MRC) National Survey of Health and Development (1946-NSHD)] and 1958 [National Child Development Study (1958-NCDS)] have been described in detail elsewhere.^20^^,^^21^ Both remain largely representative of the population from which they were drawn.^22^^,^^23^ This study consists of participants who were 60–64 years old in the 1946-NSHD (*n* = 2427) and 50 years old in the 1958-NCDS (*n* = 8674) with valid measures of PF.

### Physical functioning (outcome)

The validated Physical Component Summary (PCS) subscale of SF-36 on limitations in physical tasks due to health^24^^,^^25^ was administered once to participants at 60–64 years in 1946-NSHD and at 50 years in 1958-NCDS. As in previous work,^7^^,^^26^ poor PF was defined as the lowest, gender-specific, 10th-centile of the PCS scale within each cohort (details in Table 1).

**Table 1.** Details of physical functioning, obesity and leisure-time physical inactivity in 1946-NSHD and 1958-NCDS

Factor	Participant age (years) at ascertainment	Ascertainment method	Description
Physical functioning (outcome)	1946-NSHD: 60–64	Self-reported	The Physical Component Summary subscale of the SF-36 survey,^24^ is a validated 10-item questionnaire measuring the extent to which individuals feel they are limited in physical tasks due to their health.^24^^,^^25^^,^^27^ The scale covers a range of severe and minor limitations, including bathing or dressing, lifting, carrying groceries, climbing stairs, bending, kneeling, stooping and walking short to moderate distances. Participants were asked to respond on a three-point scale (limited ‘a lot’, ‘a little’, ‘not at all’); scores were summed and linearly transformed to a scale ranging from 0 to 100 (lower scores represent poorer PF). As in previous work,^7^^,^^26^ within each cohort, poor functioning was defined as being in the lowest, gender-specific, 10th-centile of the PF scale.
	1958-NCDS: 50		
Obesity (time-varying exposure)	1946-NSHD: 36, 43, 53 and 60–64	Height and weight were measured (1946-NSHD: all ages; 1958-NCDS: 33 years) or self-reported (1958-NCDS: 42 and 50 years)	BMI was calculated as weight/height² (kg/m²); obesity was defined as BMI ≥ 30 kg/m^2^. Three trajectories were considered as exposure groups: (i) ‘never obese’ during follow-up (i.e. 36 to 60–64 years in 1946-NSHD; 33–50 years in 1958-NCDS), (ii) ‘incident obesity’ (i.e. becoming obese; 1946-NSHD: (a) not obese at 36 years, obese 43 to 60–64 years, (b) not obese at 36 and 43 years, obese 53 to 60–64 years and (c) not obese at 36, 43 and 53 years, obese at 60–64 years; 1958-NCDS: (a) not obese at 33 years, obese 42–50 years and (b) not obese at 33 and 42 years obese at 50 years, and (iii) ‘persistent obesity’ during follow-up.
	1958-NCDS: 33, 42 and 50		
Leisure-time physical inactivity (time-varying mediator)	1946-NSHD: 36, 43, 53 and 60–64	Self-reported	In 1946-NSHD, at 36, 43, 53 and 60–64 years, participants reported how often they participated in leisure-time activity. At 36 years, participants reported the number of times they took part in 27 different sports, exercise and other leisure activities during the previous month. At 43 years, information was collected on participation in sports, exercise or other vigorous leisure activities in the previous year. At 53 and 60–64 years, participants were asked how often they participated in sports, exercise or other vigorous leisure activities during the previous 4-weeks. As in previous work,^8^^,^^28^ at each age, participants were classed as inactive if they reported no participation in leisure-time activity (i.e. participants were considered inactive if they participated in relevant activities less than once in the previous month at 36 years, per month at 43 years, and in the previous 4 weeks at 53 and 60–64 years). In 1958-NCDS, at 33, 42 and 50 years the same questions were used to ask participants about regular leisure-time activity frequency. ‘Regular’ was defined as ≥1/month for most of the year and, to aid recall, a list of example activities (of predominantly moderate or vigorous intensity, e.g. swimming, walking) was provided. Those responding affirmatively, reported activity frequency in one of six categories (every/most days, 4–5 days/week, 2–3 days/week, once a week, 2–3 times/month or less often).^29^ As in previous work,^30^^,^^31^ inactivity was defined as low activity frequency (<1/week, i.e. those reporting activity as 2–3 times/month, less often and those reporting no ‘regular’ activity).
	1958-NCDS: 33, 42 and 50		

### Obesity (time-varying exposure)

BMI (kg/m²) was calculated at 36, 43, 53 and 60–64 years in 1946-NSHD and at 33, 42 and 50 years in 1958-NCDS (Table 1). Obesity was defined as BMI ≥ 30 kg/m^2^. Three trajectories were considered as exposure groups: (i) ‘never obese’, (ii) ‘incident obesity’ (i.e. becoming obese) and (iii) ‘persistent obesity’ during follow-up (details in Table 1).

### Physical inactivity (time-varying mediator)

In 1946-NSHD, at all adult ages, participants reported how often they participated in leisure-time activity during the previous month or year (Table 1). As in previous work,^8^^,^^28^ at each age, participants were classed as inactive if they reported no leisure-time activity. In 1958-NCDS, at all adult ages, participants answered the same questions on regular leisure-time activity frequency. As in previous work,^30^^,^^31^ inactivity was defined as low activity frequency (<1/week).

### Confounding factors

Confounding factors were identified a priori, based on factors associated with obesity, inactivity and PF (details in Supplementary Table S1, available as Supplementary data at *IJE* online). These included baseline, time-invariant factors (birth to early-adulthood): gender, social class in early-life and adulthood; early adult BMI; mental health; smoking; physical activity; highest educational qualification; and illnesses in the mid-30s related to PF (arthritis/rheumatism, diabetes, heart trouble, high blood pressure, asthma). Time-varying confounding factors included smoking, mental health (i.e. depression) and, in 1958-NCDS only, self-rated health. Smoking and mental health recorded in early adulthood were treated as baseline confounders; when measured at 36, 43, 53 and 60–64 years in 1946-NSHD and at 33, 42 and 50 years in 1958-NCDS, they were treated as time-varying confounders.

## Statistical Methods

### Associations between obesity trajectories, inactivity and poor PF

In initial analyses, we used logistic regression to examine associations between obesity at each age and PF, adjusting first for baseline confounding factors and then additionally for inactivity at the same age. Analyses were repeated for inactivity at each age and PF (adjusting for baseline confounding factors and then additionally for obesity at the same age). We also used logistic regression to examine associations between obesity trajectories and PF, adjusting first for gender and then additionally for baseline confounding factors. Logistic regression is limited in its ability to (i) account for time-varying confounding and (ii) examine mediation by a time-varying factor. Hence, we adopted a counterfactual approach (described below) to address whether the association between obesity, measured at multiple time points in mid-adulthood, and subsequent PF is mediated by inactivity, measured at multiple time points.

### Mediation analysis

Using a counterfactual approach we formulated our research question in terms of a comparison of mean outcomes under alternative hypothetical scenarios. Here, we compare scenarios whereby the entire population follows certain exposure trajectories (e.g. persistently vs never obese). As we are interested in mediation by inactivity, these alternative scenarios are also specified in terms of inactivity prevalence. Given the time-varying nature of obesity, inactivity and the time-varying confounders, as depicted in Supplementary Figure S1, available as Supplementary data at *IJE* online, our targets of estimation are the randomized-interventional-analogue (randomized for short) natural effects, which invoke less stringent assumptions than natural effects.^32^ Randomized natural effects allows partitioning of the effect of obesity on poor PF into mediated (via inactivity) and non-mediated components. With randomized natural effects, the combined effect is referred to as the randomized overall (total) effect (rTE) expressed as a risk ratio (RR) that compares the predicted risk of poor PF for two alternative obesity trajectories (e.g. persistently vs never obese) that maintain the inactivity profile expected from their obesity status. Its components are the randomized natural direct effect (rNDE), i.e. the effect not mediated via inactivity, and the randomized natural indirect effect (rNIE). The rNDE for persistently vs never obese compares the predicted risk of poor PF for these two obesity trajectories, but with the prevalence of inactivity at each age set to be that of the never obese. The rNDE provides an estimate of the extent to which the risk of poor PF differs by obesity trajectories when differences in inactivity prevalence have been eliminated. The rNIE is the comparison of predicted risk of poor PF in two scenarios, e.g. where the entire population is persistently obese but the distribution of inactivity prevalence at each age is altered from the expected distribution of persistently obese to that of never obese.

We estimated these randomized effects^32^ using the parametric mediational g-formula^33^ implemented in Stata v15.1 (see Supplementary appendix, available as Supplementary data at *IJE* online, for details and code). In interpreting these estimates we invoke the assumptions of no unmeasured confounding of the exposure–outcome, mediator–outcome and exposure–mediator relationships,^33^ no measurement error and correct parametric specification of the models, in addition to the technical assumptions of no interference and causal consistency.^32^

Relationships between inactivity and obesity with PF at the last wave could be in the opposite direction to that assumed and depicted in Supplementary Figure S1, available as Supplementary data at *IJE* online. Thus, analyses were repeated omitting the last wave of data on confounders, inactivity and obesity. Analyses were also repeated separately for men and women in each cohort.

Missing data ranged from <1–19% in both cohorts. To minimize data loss and selection bias that might affect analyses, missing data were imputed via chained equations with 10 burn-in iterations.^34^ Imputation models included all model variables, plus main predictors of missingness (1946-NSHD: early adult housing tenure and childhood cognition; 1958-NCDS: childhood internalizing and externalizing behaviours and cognitive ability^23^). Logistic regressions were run across 20 imputed datasets and overall estimates obtained. For the parametric mediational g-formula, single imputation was used because standard errors were obtained via a bootstrap procedure (with 500 replications) and used to calculate 95% confidence intervals (CIs).

## Results

In both cohorts obesity prevalence increased with age, e.g. in females, from 6% at 36 years to 30% at 60–64 years in 1946-NSHD (Table 2); the most common trajectory was never obese (e.g. 64–72% in females; Table 3). In 1946-NSHD, persistent obesity was the least common trajectory (<6%), with ∼29% of males and females becoming obese at subsequent ages. In 1958-NCDS, ∼11% were persistently obese and ∼17% became obese by 50 years. Inactivity prevalence increased with age in 1946-NSHD (e.g. from 29–64% in males, Table 2), but stayed constant at ∼31% in both sexes in 1958-NCDS. At all ages, inactivity was more prevalent among the obese, e.g. in 1958-NCDS at 42 years, 40% of obese vs 32% of non-obese males were inactive (Table 3). Approximately 32% of 1946-NSHD and 19% of 1958-NCDS were ‘limited a lot’ in vigorous activities such as lifting heavy objects (Supplementary Table S2, available as Supplementary data at *IJE* online); further cohort characteristics are provided in Supplementary Table S3, available as Supplementary data at *IJE* online.

**Table 2.** Prevalence [*n* (%)] of poor physical functioning, obesity and inactivity in 1946-NSHD and 1958-NCDS. Table based on observed data; *n* varies due to missing data

	1946-NSHD			1958-NCDS		
	Age (years)	Males (*n* = 1165)	Females (*n* = 1262)	Age (years)	Males (*n* = 4173)	Females (*n* = 4501)
Poor physical functioning^a^	60–64	118 (10.1)	132 (10.5)	50	408 (9.78)	428 (9.51)
Obesity^b^						
	36	46 (4.44)	67 (5.85)	33	367 (10.6)	431 (11.1)
	43	105 (9.78)	148 (12.5)	42	667 (17.5)	613 (14.8)
	53	228 (21.6)	294 (24.8)	50	844 (24.1)	873 (23.4)
	60–64^c^	265 (27.8)	316 (29.9)			
Inactivity						
	36	306 (29.3)	465 (40.2)	33	1083 (30.4)	1184 (29.7)
	43	483 (44.6)	645 (54.1)	42	1278 (33.1)	1437 (33.7)
	53	459 (43.3)	564 (47.1)	50	1207 (29.0)	1355 (30.2)
	60–64^b^	603 (64.3)	650 (62.1)			

a Participants with Physical Component Summary subscale of the SF-36 survey scores in the lowest cohort-specific and gender-specific 10th centile (≤45 and ≤60 for males; ≤40 and ≤50 for females in 1946-NSHD and 1958-NCDS respectively) were classified as having poor physical functioning.

b Defined as body mass index  ≥30 kg/m^2^.

c 1946-NSHD only.

**Table 3.** Prevalence [*n* (%)] of obesity trajectories^a^ in mid-adulthood and inactivity at each age by obesity status at the same age^b^

		1946-NSHD					1958-NCDS			
	Age (years)	Males (*n* = 1165)		Females (*n* = 1262)		Age (years)	Males (*n* = 4173)		Females (*n* = 4501)	
Obesity trajectories^a^										
Never obese	36 to 60–64	769 (66.0)		812 (64.3)		33–50	2949 (70.7)		3247 (72.1)	
Incident obesity at:	60–64^c^	119 (10.2)		117 (9.28)						
	53	147 (12.6)		169 (13.4)		50	374 (8.96)		441 (9.79)	
	43	76 (6.48)		92 (7.29)		4y	390 (9.36)		302 (6.70)	
Persistently obese	36 to 60–64	55 (4.75)		72 (5.73)		33–50	460 (11.0)		512 (11.4)	
Inactivity		Non-obese	Obese	Non-obese	Obese		Non-obese	Obese	Non-obese	Obese
	36	326 (29.4)	19 (34.4)	459 (38.6)	49 (67.1)	33	1115 (30.0)	159 (34.6)	1169 (29.3)	180 (35.1)
	43	461 (44.1)	63 (52.8)	572 (51.7)	113 (71.9)	42	1095 (31.9)	297 (40.0)	1227 (32.1)	290 (42.5)
	53	390 (43.1)	121 (46.5)	403 (42.5)	194 (61.5)	50	840 (26.8)	372 (35.9)	898 (26.5)	460 (41.1)
	60–64^c^	521 (63.6)	238 (68.8)	523 (60.7)	277 (69.0)					

a In 1946-NSHD obesity trajectories defined as (i) never obese, (ii) persistently obese (i.e. obese at 36 years), (iii) incident obesity at 43 years (i.e. first obese at 43 years), (iv) incident obesity at 53 years (i.e. first obese at 53 years), and (v) incident obesity at 60–64 years (i.e. first obese at 60–64 years); in 1958-NCDS obesity trajectories defined as: (i) never obese, (ii) persistently obese (i.e. obese at 33 years), (iii) incident obesity at 42 years (i.e. first obese at 42 years), (iv) incident obesity at 50 years (i.e. first obese at obese at 50 years). Trajectories are defined in this way because it was rare to move from being obese to non-obese, e.g. in 1946-NSHD prevalence of obesity at 36  and 43 years but not thereafter: < 0.05% in both males and females; prevalence of obesity at 36, 43, 53 but not 60–64 years: < 0.15% in both males and females; in 1958-NCDS, prevalence of obesity at 33 and 42 years but not at 50 years: < 1% in both males and females; prevalence of obesity at 33 years but not thereafter: 1.41% (males)/1.30% (females).

b Table averaged across 20 imputed datasets.

c 1946-NSHD only.

### Associations between obesity trajectories, inactivity and poor physical functioning

Obesity at each age was associated with poor PF: adjusted odds ratios (ORs_adjusted_) varied from 1.75 (95% CI: 1.24, 2.49) for 53 year obesity to 2.39 (1.57, 3.64) for 43 year obesity in 1946-NSHD and 1.53 (1.17, 2.01) for 33 year obesity to 1.63 (1.34, 1.99) for 50 year obesity in 1958-NCDS (Table 4); associations were little affected after further adjustment for concurrent inactivity. Similarly, inactivity at each age was associated with poor PF; adjustment for concurrent obesity slightly attenuated relationships. In 1946-NSHD, the OR_adjusted_ of incident obesity at 43 years vs never obese (36 to 60–64 years) on poor PF was 2.71 (1.63, 4.48), for incident obesity at 53 years it was 1.65 (1.06, 2.56); the OR_adjusted_ of persistent obesity was 4.07 (2.16, 7.66) (Table 4). In 1958-NCDS, patterns of association were similar albeit weaker in magnitude.

**Table 4.** Odds ratios (95% CI) for poor physical functioning of obesity and inactivity at each age and obesity trajectories during follow-up^a^; table based on imputed data

	Poor physical functioning					
	1946-NSHD (at 60–64 years)			1958-NCDS (at 50 years)		
Obesity	Age (years)	Model 1^b^	Model 2^c^	Age (years)	Model 1^b^	Model 2^c^
	36	2.29 (1.30,4.02)	2.17 (1.23,3.81)	33	1.53 (1.17,2.01)	1.52 (1.16,2.00)
	43	2.39 (1.57,3.64)	2.28 (1.49,3.49)	42	1.56 (1.25,1.94)	1.51 (1.21,1.89)
	53	1.75 (1.24,2.49)	1.69 (1.18,2.42)	50	1.63 (1.34,1.99)	1.53 (1.26,1.87)
	60–64^d^	2.08 (1.49,2.91)	2.08 (1.48,2.90)			
Inactivity						
	36	1.83 (1.37,2.44)	1.79 (1.34,2.40)	33	1.26 (1.06,1.50)	1.25 (1.05,1.49)
	43	1.97 (1.44,2.70)	1.91 (1.39,2.63)	42	1.51 (1.29,1.77)	1.49 (1.27,1.74)
	53	2.54 (1.84,3.52)	2.50 (1.81,3.46)	50	2.27 (1.95,2.65)	2.22 (1.91,2.59)
	60–64^d^	2.33 (1.56,3.47)	2.32 (1.55,3.47)			
Obesity trajectories		Model A^e^	Model B^f^		Model A^e^	Model B^f^
Never obese	36 to 60–64	Reference	Reference	33–50	Reference	Reference
Incident obesity at:	60–64^d^	2.07 (1.28, 3.37)	1.64 (0.98, 2.73)			
	53	2.20 (1.47, 3.29)	1.65 (1.06, 2.56)	50	1.70 (1.33,2.17)	1.46 (1.12,1.89)
	43	4.06 (2.64, 6.23)	2.71 (1.63, 4.48)	42	2.17 (1.71,2.76)	1.69 (1.29,2.21)
Persistently obesity	36 to 60–64	6.44 (4.16, 9.96)	4.07 (2.16, 7.66)	33–50	2.84 (2.34,3.45)	1.94 (1.45,2.59)

a Follow-up refers to ages 36 to 60–64 years in 1946-NSHD and 33–50 years in 1958-NCDS; obesity trajectories are as defined in Table 3 footnotes.

b Model 1: adjusted for gender, social class in early-life and adulthood; early adult BMI; mental health; smoking; physical activity (1958-NCDS only); highest educational qualification and illnesses: arthritis/rheumatism; diabetes; heart trouble; high blood pressure; and asthma; see Supplementary Table S1, available as Supplementary data at *IJE* online, for further details.

c Model 2 adjusted for Model 1 factors and obesity/inactivity at the same age (as appropriate).

d 1946-NSHD only.

e Model A: adjusted for gender.

f Model B: additionally adjusted for social class in early-life and adulthood; early adult BMI; mental health; smoking; physical activity (1958-NCDS only); highest educational qualification and illnesses: arthritis/rheumatism; diabetes; heart trouble; high blood pressure; and asthma; see Supplementary Table S1, available as Supplementary data at *IJE* online for further details.

### Mediation analysis

Table 5 presents the estimated rTE and its partition into rNDE and rNIE (through inactivity) for different obesity trajectories.

**Table 5.** Randomized total, natural direct and natural indirect effects (risk ratios, 95% CIs) of incident obesity at selected ages and of persistent obesity vs never obese during follow-up^a^ on poor physical functioning at 60–64/50 years (mediated by time-varying inactivity)^b^

	1946-NSHD				1958-NCDS		
	Incident obesity at 60–64 years^c^	Incident obesity at 53 years	Incident obesity at 43 years	Persistent obesity (from 36 years)	Incident obesity at 50 years^c^	Incident obesity at 42 years	Persistent obesity (from 33 years)
Randomized total effect	1.32 (0.75, 1.88)	1.53 (0.91, 2.15)	2.32 (1.13, 3.51)	2.91 (1.14, 4.69)	1.14 (0.94, 1.34)	1.22 (0.96, 1.48)	1.53 (1.12, 1.93)
Randomized natural direct effect (not via physical inactivity)		1.50 (0.89, 2.11)	2.27 (1.13, 3.41)	2.84 (1.16, 4.51)		1.20 (0.94, 1.45)	1.49 (1.09, 1.88)
Randomized natural indirect effect (via physical inactivity)		1.02 (0.99, 1.04)	1.02 (0.97, 1.07)	1.03 (0.96, 1.10)		1.02 (1.01, 1.03)	1.03 (1.01, 1.05)

a Follow-up refers to ages 36 to 60–64 years in 1946-NSHD and 33 to 50 years in 1958-NCDS.

b Adjusted for: (i) baseline confounders: gender, early-life and adult social class, early adult BMI, mental health, smoking, physical activity (1958-NCDS only), highest educational qualification, illnesses: arthritis/rheumatism, diabetes, heart trouble, high blood pressure and asthma; and (ii) time-varying confounders: smoking, depression and self-rated health (1958-NCDS only), see Supplementary Table S1, available as Supplementary data at *IJE* online, for details.

c For incident obesity at 60–64 years in 1946-NSHD and at 50 years in 1958-NCDS, the randomized total effect is not mediated by inactivity: we assume inactivity precedes obesity (i.e. there is no measure of inactivity between obesity and physical functioning), see Supplementary Figure S1 and appendix, available as Supplementary data at *IJE* online, for details.

*Incident vs. never obese*: in 1946-NSHD, the estimated rTE of incident obesity at 43 years vs never obese on poor PF, expressed as a RR was 2.32 (1.13, 3.51), and for incident obesity at 53 years was 1.53 (0.91, 2.15) (Table 5). When partitioned, the estimated rNDE-RRs of incident obesity at 43 and 53 years were 2.27 (1.13, 3.41) and 1.50 (0.89, 2.11) respectively; the estimated rNIE-RRs were 1.02 (0.97, 1.07) and 1.02 (0.99, 1.04). In 1958-NCDS, patterns of association were similar, although weaker in magnitude; e.g. for 42 years incident obesity, the rTE-RR of poor PF at 50 years was 1.22 (0.96, 1.48) and rNDE-RR was 1.20 (0.94, 1.45).

*Persistently vs. never obese*: in 1946-NSHD, the estimated rTE-RR of persistent obesity (vs never obese) on poor PF was 2.91 (1.14, 4.69), with estimated rNDE-RR of 2.84 (1.16, 4.51) and rNIE-RR of 1.03 (0.96, 1.10) (Table 5). In 1958-NCDS, patterns of association were similar albeit weaker.

Results were similar to those presented above when information on inactivity, obesity and confounding factors at the final time-point were omitted (Supplementary Table S4, available as Supplementary data at *IJE* online). When analyses were stratified by gender, associations were broadly similar for men and women in 1958-NCDS; in 1946-NSHD associations appeared stronger for men, although CIs were wide (Supplementary Tables S5 and S6, available as Supplementary data at *IJE* online).

## Discussion

In two general population cohorts with lifetime follow-up in Britain, we identified two important and consistent findings. First, obesity from the mid-30s was associated with poor PF in late middle-age, with the odds for poor PF in the early 60s being ∼2-fold higher for obesity from 36 years onwards. Importantly, more detrimental associations were observed for longer duration of obesity, with onset from as early as the mid-30s having an ∼3-fold higher risk of poor PF at 60–64 years, and a 2-fold higher risk associated with onset from the mid-40s. Second, the indirect effect of obesity on poor PF through inactivity was small, e.g. the overall increased risk of persistent obesity from the mid-30s on poor PF at 50 years was 53%; after considering mediation by adult inactivity, the increased risk was 49%. Thus, our findings suggest an influence of obesity on later PF that is largely via pathways other than leisure-time inactivity, even though inactivity prevalence was consistently higher among the obese vs non-obese at all ages in our study.

A key study strength is the inclusion of two populations designed to be nationally representative with prospective and comparable measures of obesity and inactivity over decades of adult life. Examining associations in two birth cohorts has several advantages including (to the extent that study design allows), standardization of research aims and analytic approaches, allowing us to determine whether findings are similar across generations. Importantly, our mediation analysis used appropriate statistical methods that allow for bi-directional relationships between obesity and inactivity over the life-course. Thus, we avoid potential biases inherent in simpler methods and more accurately reflect processes in the real world. We also had rich prospectively recorded data for potential confounding factors from earlier life and that vary over mid-adulthood. Nonetheless, study weaknesses are acknowledged. Outcomes were recorded at different ages in the two cohorts. Some measures (e.g. inactivity) were self-reported and misclassification of an individual’s status is possible. However, the measures of obesity, inacitvity and PF used all predict mortality,^35–37^ and self-report is a practical means of obtaining information in large scale studies such as those examined here. Our PF measure has been widely used in general and older populations,^24^ and has been validated against objective assessments of physical performance.^25^ Although common, obesity indicated by BMI may not adequately measure body fatness. The frequency-based leisure-time inactivity measures used are crude, do not encompass occupational, active travel or domestic physical activity, and vary slightly between cohorts (see Table 1). However despite limitations, our focus on inactivity is important given evidence that even low activity levels (i.e. avoidance of inactivity) protects against mortality.^38^^,^^39^ PF at earlier ages was not ascertained, although it may influence subsequent inactivity and obesity. However, we accounted for several conditions in the mid-30s, e.g. arthritis/rheumatism that could affect PF and, in 1958-NCDS, for repeated self-rated general health. We acknowledge that relationships between inactivity and obesity with PF at the last wave could be in the opposite direction to that assumed in Supplementary Figure S1, available as Supplementary data at *IJE* online, however, analysis that excluded inactivity and obesity measures at the same sweep as PF showed similar results to those reported. Our mediation analysis is computationally intensive and may be prone to bias due to model misspecification (e.g. ordering at each age of confounding factors, inactivity, obesity; particularly the assumption that inactivity occurs prior to obesity). Moreover, alternative related questions remain to be examined, such as whether increasing physical activity over time is associated with subsequent PF. The mediation analysis relies on several other unverifiable assumptions, including no unmeasured confounding,^33^ no interference and causal consistency. To attempt to meet the assumption of no unmeasured confounding, we controlled for several likely confounding factors, however we acknowledge the possibility of residual confounding. The no interference assumption would not be satisfied if, e.g. obesity status of one individual influenced the inactivity and PF of another individual. However, because study participants are located across a wide geographical area, it is plausible that the assumption of no interference is met. Causal consistency, would imply, e.g. that intervening on inactivity and freely choosing that level of inactivity would have the same impact on PF. Although both the simple and mediation analyses rely on the assumption of no unmeasured confounding, the simple analysis does not account for time-varying confounding, whereas the mediation analysis is computationally intensive. Conclusions regarding obesity trajectories and poor PF associations were broadly similar using both methods. Results were also consistent with those found in sensitivity analyses, together providing reassurance regarding the robustness of our findings. Finally, although our study populations were selected to be nationally representative at birth, and remain so in many respects,^22^^,^^23^ loss to follow-up due to death and non-participation has occurred. However, we maximized available data by including participants with a valid measure of PF and avoided sample reductions due to missing information by using imputation.

Our study is important given that poor PF at the life-stage examined is associated with subsequent adverse health outcomes, including premature death.^40^ As most previous studies of obesity, (in)activity and PF do not investigate repeat measures in a life-course context, we contribute to the field by, first, establishing associations between adult obesity and PF and second, illuminating the potential role of inactivity in this relationship. With respect to the former, consistently across cohorts, we show that obesity at any adult age from the mid-30s was associated with poor PF in late middle-age. More detrimental associations were observed for longer duration of obesity, e.g. in 1958-NCDS, the RR for rTE increased from 1.14 to 1.53 with decreasing age of obesity onset, in accord with previous findings in these cohorts^7^^,^^41^ and with the broader literature showing adult obesity^4–6^ and longer obesity duration^5^^,^^6^ to be associated with poor PF from mid-adulthood onwards. In relation to obesity duration, we showed previously that childhood onset was associated with higher BMI in mid-adulthood, which in turn might underlie the higher risk of concurrent poor PF.^7^ Excess body weight is also implicated by studies of intentional weight loss, which is associated with improvements in PF.^42^ A noteworthy observation is that associations between obesity trajectories and poor PF were stronger in 1946-NSHD compared with 1958-NCDS, e.g. the rTE-RR for always obese (from the mid-30s) on poor functioning was 2.91 in 1946-NSHD vs 1.53 in 1958-NCDS. This could be a consequence of the older age of 1946-NSHD participants at PF assessment, which is associated with greater functional limitations and also with the possibility of longer duration of obesity compared with 1958-NCDS participants. The differing magnitude of association across generations is interesting given that obesity from the mid-30s was more common among those born in 1958 than in 1946 (see Tables 2 and 3). Once comparable measures are available at overlapping ages in different birth cohorts, future research should examine birth cohort differences in associations between obesity trajectories and poor PF at similar ages.

Our study is unique in examining time-varying inactivity as a potential intermediary of the obesity–poor PF relationship. The counterfactual framework used sheds light on likely effects of a hypothetical intervention to reduce inactivity prevalence among the obese (to that of the non-obese) on the obesity trajectories–PF association. If underlying assumptions are met, our effect estimates could have a causal interpretation.^43^ Using this approach, we found the effect of obesity on poor PF mediated by inactivity to be small. This finding was consistent across two cohorts and is striking, given that whereas the rTE of obesity on functioning was stronger in 1946-NSHD than 1958-NCDS, the rNIEs (via inactivity) were markedly similar. Our findings suggest a small indirect effect of obesity on poor PF through inactivity, and a larger influence of obesity on PF via alternative pathways. There are several possible explanations for a small indirect effect via inactivity. Our inactivity measure may not sufficiently capture activity type, which may be important, given evidence linking strength training to improved PF;^44^ although other components of activity such as intensity, may also be relevant. Our binary inactivity measure may be too crude to detect a mediating effect^45^ and thus further studies of mediation by time-varying activity are required that account for activity type, duration, intensity and frequency. Other factors may mediate between obesity and PF, e.g. obesity causally influences inflammation as indicated by biomarkers such as C-reactive protein,^46^ which in turn has been postulated to lead to muscle weakness and sarcopenia.^47^ Accordingly, longer obesity duration and associated higher adiposity levels may result in a persistently elevated inflammatory response, leading to decreased functional capacity even in mid-life.

## Conclusion

In conclusion, we found obesity at any adult age (from the mid-30s) was adversely associated with PF, with more detrimental associations observed for longer duration of obesity. Such observations highlight that opportunities for successful interventions to avert obesity need to start at young ages and continue throughout the lifespan. Moreover, despite the relatively young age of our populations, detrimental associations for obesity from the mid-30s were observed, e.g. with 53% higher risk of poor PF at 50 years. This is important because, given current trends,^11^ higher proportions of younger generations will be obese from at least as early as mid-adulthood. Our findings therefore suggest that much of the future adult population will be at risk of poor PF before reaching older ages. We also found that inactivity played a small mediating role in the obesity–PF association, suggesting that although avoidance of inactivity might be expected to reduce some of the impact of obesity on later PF, much of its effect is likely to be via other pathways. In light of ageing of the global population,^1^ coupled with high obesity prevalence,^11^ future studies are warranted to investigate underlying mechanisms linking obesity to poor PF. In summary, our findings together with other evidence, stress the importance of preventing and delaying onset of obesity to protect against poor PF.

## Supplementary Data

Supplementary data are available at *IJE* online.

## Funding

This work was supported by the Department of Health Policy Research Programme through the Public Health Research Consortium (PHRC) and supported by the National Institute for Health Research Biomedical Research Centre at Great Ormond Street Hospital for Children NHS Foundation Trust and University College London. S.M.P.P. is funded by a UK Medical Research Council (MRC) Career Development Award (ref: MR/P020372/1). The views expressed in the publication are those of the authors and not necessarily those of the funding bodies. Information about the wider programme of the PHRC is available from http://phrc.lshtm.ac.uk. The MRC NSHD and R.H. were supported by the UK MRC (MC_UU_12019/1, MC_UU_12019/2). R.H. is Principal Investigator of the CLOSER consortium which was funded by the Economic and Social Research Council (ESRC) and the MRC from 2012 to 2017. The initial five year grant has been extended by the ESRC (award reference: ES/K000357/1).The funders had no input into study design; data collection, analysis, and interpretation; in the writing of the report; and in the decision to submit the article for publication. Researchers were independent of influence from study funders.

## Supplementary Material

dyaa014_Supplementary_Data
