# Supplementary material for: Adult obesity and mid-life physical functioning in two British birth cohorts: investigating the mediating role of physical inactivity
Source: Int J Epidemiol. 2020 Mar 6;49(3):845–56. doi: 10.1093/ije/dyaa014 (PMC7394955; doi:10.1093/ije/dyaa014)
Supplement: dyaa014_Supplementary_Data [file dyaa014_supplementary_data.docx]

**Supplementary Material**

**
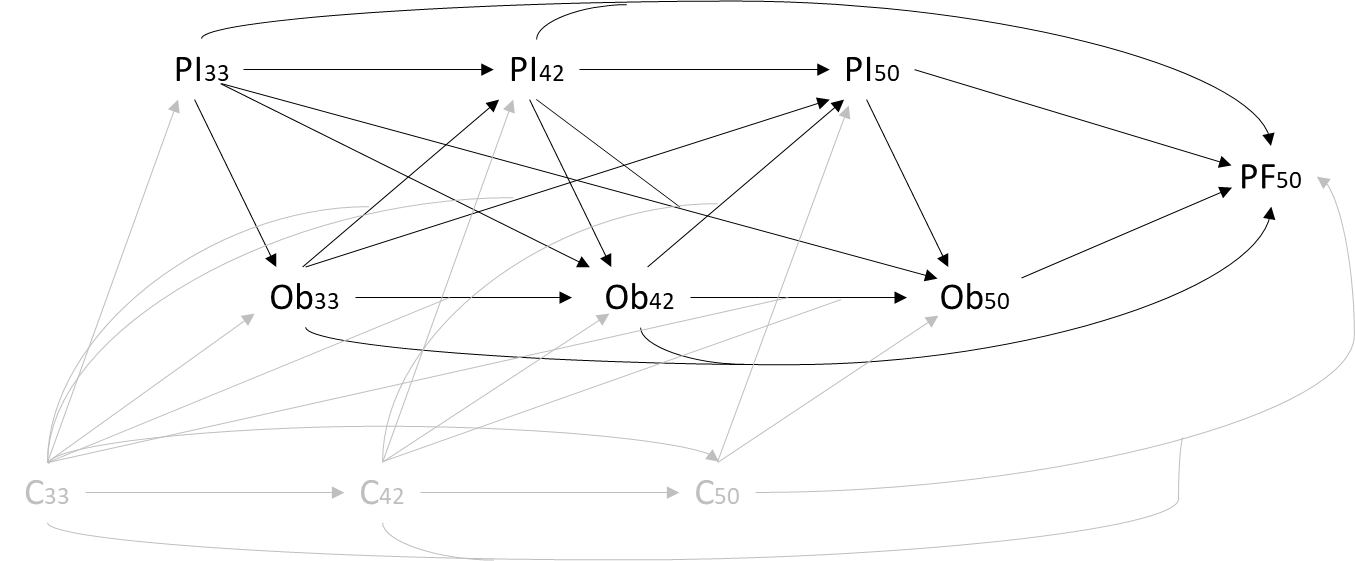
Figure S1**: Representation of obesity (Ob, exposure), physical inactivity (PI, mediator) and confounding factors (C) that vary over time in relation to physical functioning (PF, outcome) in 1958-National Child Development Study (NCDS).

Diagram represents 1958-NCDS, similar diagram with ages 36y, 43y, 53y and 60-64y would represent 1946-NSHD. For Lin’s algorithm (Epidemiology 28(2): 266-274) we let t=0, 1, 2 correspond to ages 33y, 42y and 50y respectively in 1958-NCDS and let t=0, 1, 2, 3 correspond to ages 36y, 43y, 53y and 60-64y respectively in 1946-National Survey for Health and Development (NSHD), see appendix for details.

**Table S1: Covariate details and summary of covariate models**

| Variable | Type of model when used as a dependent variable | Functional form when used as a predictor |
| --- | --- | --- |
| *Time-invariant (reporting age in 1946-NSHD/1958-NCDS)* |  |  |
| Gender (birth) | Not predicted | Two categories^d^ |
| Social class (childhood/birth) | Not predicted | Four categories^a^ |
| Social class (53y/33y) | Not predicted | Four categories^a^ |
| BMI (26/23y) | Not predicted | Linear |
| Mental health^h^ (13-15/23y) | Not predicted | Linear |
| Smoking (26/23y) | Not predicted | Three categories^b^ |
| Physical activity^c^ (NA/23y) | Not predicted | Four categories^a^ |
| Educational qualification (26/33y) | Not predicted | Four categories^a^ |
| Arthritis/rheumatism (36/33y) | Not predicted | Two categories^g^ |
| Diabetes (36/33y) | Not predicted | Two categories^g^ |
| Heart trouble (36/33y) | Not predicted | Two categories^g^ |
| High blood pressure (36/33y) | Not predicted | Two categories^g^ |
| Asthma (36/33y) | Not predicted | Two categories^g^ |
| *Time-varying* |  |  |
| Smoking | Multinomial logistic^f^ | Three categories^b^ |
| Depression ^h^ | Logistic^f^ | Two categories^d^ |
| Self-rated health^c^ | Multinomial logistic^f^ | Four categories^a^ |
| Physical inactivity^e^ | Logistic | Two categories^d^ |
| Obesity | Not predicted | Two categories^d^ |

NSHD: Medical Research Council National Survey for Health and Development; NCDS: National Child Development Survey

^a^Social class categories (according to the Registrar General’s Classification) are: professional/managerial, skilled non-manual, skilled manual, semiskilled and unskilled manual. Physical activity categories are: not at all in the last 4 weeks, 1-3 times in the last 4 weeks, once/twice a week and 3+ times a week. Educational qualification categories are: <O-levels, O-levels, A-levels, degree level. Self-rated health categories are: poor, fair, good/very good, excellent.

^b^Smoking categories are: never smoker, ex-smoker, current-smoker

^c^1958-NCDS only; for details on 23y physical activity see Parsons TJ et al *Med Sci Sports Exerc* 2006; 38(3): 547-54

^d^Categories are no/yes (for gender: males, females)

^e^defined as: no participation in leisure-time activity in 1946-NSHD; participation <1/week or no regular activity in 1958-NCDS. See methods and Table 1 for details.

^f^not modelled as a dependent variable at t=0

^g^in 1958-NCDS categories are no/yes, in 1946-NSHD illness (arthritis/rheumatism, diabetes, heart trouble, high blood pressure, asthma) are coded together as a range: 0, 1, 2+

^h^ In 1946-NSHD at 13y and 15y teacher ratings of behaviour and temperament were obtained using a forerunner of the Rutter A scale; factor scores at 13y and 15y were summed to create scales representing a dimension of emotional problems, and were standardised to a mean of 0 and SD of 1 (for further details on mental health and depression in 1946-NSHD see James SN et al *J Affect Disord* 2018; 241: 348-55). In 1958-NCDS, depressive symptoms were measured at 23y using 15 yes/no items from the Psychological subscale of the Malaise Inventory assessing common symptoms of depression and anxiety (for further details on mental health and depression in 1958-NCDS see Pinto Pereira SM et al *JAMA Psychiat* 2014; 71(12): 1373-80).

**Table S2: Physical Component Summary subscale questions: prevalence (N(%)) “limited a lot”* at 60-64y (1946-NSHD) and 50y (1958-NCDS)**

|  | 1946-NSHD | 1958-NCDS |
| --- | --- | --- |
| Vigorous activities, such as running, lifting heavy objects, participating in strenuous sports | 748 (31.7) | 1637 (18.9) |
| Moderate activities, such as moving a table, pushing a vacuum cleaner, bowling, or playing golf | 149 (6.18) | 457 (5.27) |
| Lifting or carrying groceries | 160 (6.73) | 429 (4.95) |
| Climbing several flights of stairs | 265 (11.0) | 590 (6.80) |
| Climbing one flight of stairs | 117 (4.88) | 341 (3.93) |
| Bending, kneeling or stooping | 258 (10.7) | 668 (7.70) |
| Walking more than one mile | 276 (11.5) | 615 (7.09) |
| Walking half a mile | 196 (8.16) | 490 (5.65) |
| Walking 100 yards | 99 (4.13) | 327 (3.77) |
| Bathing or dressing yourself | 62 (2.57) | 252 (2.91) |

NSHD: Medical Research Council National Survey for Health and Development; NCDS: National Child Development Survey

N varies due to missing data

*cohort members were asked “Does your health limit you in these activities? If so, how much?” Responses were: yes, limited a lot; yes, limited a little; no, not limited at all

**Table S3: Covariate distribution in 1946-NSHD and 1958-NCDS: N(%)**

| Variable (reporting age in 1946-NSHD/1958-NCDS) | 1946-NSHD | 1958-NCDS |
| --- | --- | --- |
| Gender (birth) |  |  |
| Male | 1165 (48.0) | 4173 (48.1) |
| Social class (childhood/birth) |  |  |
| Professional/managerial | 659 (28.5) | 1664 (19.7) |
| Skilled non-manual | 396 (17.2) | 874 (10.4) |
| Skilled manual | 702 (30.4) | 4031 (47.8) |
| Semiskilled/unskilled manual | 552 (23.9) | 1862 (22.1) |
| Social class (53y/33y) |  |  |
| Professional/managerial | 1103 (45.8) | 2751 (38.7) |
| Skilled non-manual | 571 (23.7) | 1793 (25.2) |
| Skilled manual | 380 (15.8) | 1322 (18.6) |
| Semiskilled/unskilled manual | 355 (14.7) | 1251 (17.6) |
| BMI (26y/23y)* | 22.8 (3.0) | 22.9 (3.1) |
| Smoking (26y/23y) |  |  |
| Never | 699 (35.4) | 4000 (54.2) |
| Ex-smoker | 540 (27.3) | 753 (10.2) |
| Current | 738 (37.3) | 2626 (35.6) |
| Physical activity (NA/23y) |  |  |
| not at all in the last 4 weeks |  | 3734 (50.4) |
| 1-3 times in the last 4 weeks |  | 1218 (16.5) |
| once/twice a week |  | 1404 (19.0) |
| 3+ times a week |  | 1047 (14.1) |
| Educational qualification (26y/33y) |  |  |
| <O-levels | 922 (40.2) | 1581 (21.3) |
| O-levels | 488 (21.3) | 2530 (34.1) |
| A-levels | 623 (27.2) | 2241 (30.2) |
| Degree level | 261 (11.4) | 1078 (14.5) |
| Arthritis/rheumatism (36y/33y) | 180 (8.20) | 1128 (14.9) |
| Diabetes (36y/33y) | 7 (0.32) | 75 (1.00) |
| Heart trouble (36y/33y) | 18 (0.82) | 135 (1.79) |
| High blood pressure (36y/33y) | 67 (3.04) | 611 (8.11) |
| Asthma (36y/33y) | 60 (2.72) | 2131 (28.2) |

NSHD: Medical Research Council National Survey for Health and Development; NCDS: National Child Development Survey

N varies due to missing data

*kg/m^2^; Mean(SD)

**Table S4^¥^: Randomised total, natural direct and natural indirect effects (Risk Ratios, 95% CIs) of incident obesity at selected ages and of persistent obesity vs. never obese during follow-up* on poor physical functioning at 60-64y/50y (mediated by time-varying inactivity)****

|  | 1946-NSHD | | | 1958-NCDS | |
| --- | --- | --- | --- | --- | --- |
|  | incident obesity at 53y | incident obesity at 43y | persistently obese (from 36y) | incident obesity at 42y | persistently obese (from 33y) |
| Randomised total effect | 1.37 (0.97,1.78) | 2.28 (1.38,3.18) | 3.17 (1.74,4.60) | 1.10 (0.89,1.31) | 1.49 (1.14,1.83) |
| Randomised natural direct effect  (not via physical inactivity) | 1.37 (0.97,1.78) | 2.24 (1.36,3.11) | 3.08 (1.71,4.45) | 1.10 (0.89,1.31) | 1.48 (1.14,1.82) |
| Randomised natural indirect effect  (via physical inactivity) |  | 1.02 (0.98,1.06) | 1.03 (0.97,1.09) |  | 1.01 (0.99,1.02) |

NSHD: Medical Research Council National Survey for Health and Development; NCDS: National Child Development Survey

^¥^Analysis ignores final sweep of data on confounders, inactivity and obesity, see text for details

*follow-up refers to ages 36y to 60-64y in 1946-NSHD and 33y to 50y in 1958-NCDS

**adjusted for: (i) time-invariant confounders: gender, early-life and adult social class, early adult BMI, mental health, smoking, physical activity (1958-NCDS only), highest educational qualification, illnesses: arthritis/rheumatism, diabetes, heart trouble, high blood pressure and asthma; and (ii) time-varying confounders: smoking, depression and self-rated health (1958-NCDS only), see Supplementary Table 1 for details

**Table S5: Randomised total, natural direct and natural indirect effects (Risk Ratios, 95% CIs) of incident obesity at selected ages and of persistent obesity vs. never obese during follow-up* on poor physical functioning at 60-64y stratified by gender in 1946-NSHD (mediated by time-varying inactivity)****

|  | incident obesity at 60-64y^¥^ | | incident obesity at 53y | | incident obesity at 43y | | persistently obese (from 36y) | |
| --- | --- | --- | --- | --- | --- | --- | --- | --- |
|  | Males | Females | Males | Females | Males | Females | Males | Females |
| Randomised total effect | 2.00 (1.21,2.78) | 1.12 (0.73,1.52) | 2.38 (1.41,3.35) | 1.16 (0.79,1.52) | 3.60 (1.76,5.44) | 1.59 (0.85,2.33) | 5.36  (2.47,8.25) | 1.48 (0.48,2.49) |
| Randomised natural direct effect  (not via physical inactivity) |  |  | 2.32 (1.37,3.27) | 1.15 (0.79,1.51) | 3.52 (1.70,5.33) | 1.53 (0.84,2.23) | 5.23  (2.43,8.04) | 1.41 (0.47,2.36) |
| Randomised natural indirect effect  (via physical inactivity) |  |  | 1.02  (0.99,1.06) | 1.01 (0.99,1.03) | 1.02  (0.95,1.09) | 1.04 (0.99,1.08) | 1.02  (0.93,1.12) | 1.05 (0.98,1.12) |

NSHD: Medical Research Council National Survey for Health and Development

*follow-up refers to ages 36y to 60-64y

**adjusted for: (i) time-invariant confounders: early-life and adult social class, early adult BMI, mental health, smoking, highest educational qualification, illnesses (range: 0, 1, 2+): arthritis/rheumatism, diabetes, heart trouble, high blood pressure and asthma; and (ii) time-varying confounders: smoking and depression, see Supplementary Table 1 for details

^¥^ for incident obesity at 60-64y the randomised total effect is not mediated by inactivity: we assume inactivity precedes obesity (i.e. there is no measure of inactivity between obesity and physical functioning), see Supplementary Figure 1 and appendix for details

**Table S6: Randomised total, natural direct and natural indirect effects (Risk Ratios, 95% CIs) of incident obesity at selected ages and of persistent obesity vs. never obese during follow-up* on poor physical functioning at 50y stratified by gender in 1958-NCDS (mediated by time-varying inactivity)****

|  | incident obesity at 50y^¥^ | | incident obesity at 42y | | persistently obese (from 33y) | |
| --- | --- | --- | --- | --- | --- | --- |
|  | Males | Females | Males | Females | Males | Females |
| Randomised total effect | 1.34  (1.03,1.65) | 1.13  (0.83,1.43) | 1.04  (0.71,1.38) | 1.33  (0.86,1.80) | 1.44  (0.93,1.94) | 1.64  (0.98,2.29) |
| Randomised natural direct effect  (not via physical inactivity) |  |  | 1.03  (0.70,1.36) | 1.30  (0.85,1.76) | 1.41  (0.91,1.90) | 1.59  (0.95,2.22) |
| Randomised natural indirect effect  (via physical inactivity) |  |  | 1.02  (0.99,1.04) | 1.02  (0.99,1.04) | 1.02  (0.99,1.05) | 1.03  (0.99,1.06) |

NCDS: National Child Development Survey

*follow-up refers to ages 33y to 50y

**adjusted for: (i) time-invariant confounders: early-life and adult social class, early adult BMI, mental health, smoking, physical activity, highest educational qualification, binary illnesses indicators: arthritis/rheumatism, diabetes, heart trouble, high blood pressure and asthma; and (ii) time-varying confounders: smoking, depression and self-rated health, see Supplementary Table 1 for details

^¥^ for incident obesity at 50y in 1958-NCDS, the randomised total effect is not mediated by inactivity: we assume inactivity precedes obesity (i.e. there is no measure of inactivity between obesity and physical functioning), see Supplementary Figure 1 and appendix for details

**Appendix**

*Representation of confounding factors, physical inactivity and obesity that vary over time in relation to physical functioning*

Supplementary Figure 1 shows the presumed relationships between confounding factors (e.g. smoking, C), physical inactivity (mediator, PI) and obesity (exposure, Ob) with physical functioning (PF). We let t=0, 1, 2 and 3 correspond to ages 36y, 43y, 53y and 60–64y respectively for 1946-NSHD and t=0, 1, and 2 correspond to ages 33y, 42y and 50y respectively for 1958-NCDS. At each time t, a person can be either obese (Ob_t_=1) or non-obese (Ob_t_=0). Physical inactivity is also measured at each time t (PI_t_=1: inactive; PI_t_ =0: not inactive); we assume at each time t, PI_t_ precedes Ob_t_. C_t_ is the vector of potential confounding factors at time t; time-invariant confounding factors are included in C_0_. We assume at each time t, C_t_ proceeds both PI_t_ and Ob_t_.

*Mediation analysis using the parametric mediational g-formula: details*

We adopted the counterfactual approach reported by Lin et al^1^. In counterfactual-based methods, observed data are used to predict physical functioning (outcome), physical inactivity (time-varying mediator) and time-varying confounders that would have been observed under the obesity trajectories described in the main text. We specify models for physical functioning, as well as for inactivity and confounders at each time point. We use current and all past covariates as predictors (illustrated in Supplementary Figure 1). For example, in 1958-NCDS, we regress PF on Ob_2_, PI_2_, C_2_, Ob_1_, PI_1_, C_1_, Ob_0_, PI_0_ and C_0_. For t=0, 1, 2 we regress PI_t_ on C_t_, Ob_t-1_, PI_t-1_, C_t-1_,…, Ob_0_, PI_0_ and C_0_ and regress C_t_ on Ob_t-1_, PI_t-1_, C_t-1_,…, Ob_0_, PI_0_ and C_0_, in each case using appropriate regression models (see Supplementary Table 1). Next, we predict time-varying inactivity (i.e. the time-varying mediator) and physical functioning (i.e. the outcome) under the time-varying obesity trajectories of interest. Finally, we estimate the average potential outcome under each scenario and use them to calculate the randomised total, natural direct and natural indirect effects. Our implementation of Lin’s algorithm^1^ (see statistical code below) starts with the expansion of our original data 100 times (in order to minimise Monte Carlo error in the simulations), whilst retaining an identifier of the original records (to ensure that the model parameters are estimated using the original data, rather than the expanded data).

*STATA code implementing Lin’s algorithm^1^*

Code below relates to the 1958-NCDS analysis where t=0, 1, 2 corresponds to ages 33y, 42y and 50y respectively. Variables are labelled such that: y refers to poor physical functioning (as defined in the main text); a0, a1, a2 refers to obesity at each age; m0, m1, m2 refers to inactivity at each age; l10, l11, l12 refers to self-rated health at each age; l20, l21, l22 refers to smoking at each age; l30, l31, l32 refers to depression at each age. Variables c1_0 to c13_0 refer to time-invariant confounders (i.e. 23y smoking, 33y education, 33y arthritis/rheumatism, 33y diabetes, 33y heart trouble, 33y high blood pressure, 33y asthma, sex, 33y social class, 23y BMI, 23y physical activity, social class at birth and 23y mental health, see Supplementary Table 1 for details).

The code is run in a program (called MM_tvar) that first performs the imputation using iterative chain equations; code for the Monte Carlo simulation and Lins’ algorithm are detailed below.

cap program drop MM_tvar

cap program define MM_tvar, rclass

preserve

* single imputation step using chained equations with 10 burn-in iterations

**********************************************************************************************

*MONTE CARLO STEP

**********************************************************************************************

expand 100, gen(temp)

tab temp

gen original = 1 if temp==0

tab original

drop temp

**********************************************************************************************

*Lin’s algorithm

**********************************************************************************************

*comparing (a) never obese (a=0 always) to (b) always obese (a=1 always) (c) become obese 42y (0,1,1) (d) become obese at 50y (0,0,1)

*******************

****PART 1 AND 2a

*******************

*l11

mlogit l11 i.a0 i.m0 i.c1_0 i.c2_0 i.c3_0 i.c4_0 i.c5_0 i.c6_0 i.c7_0 i.c8_0 i.c9_0 c10_0 i.c11_0 i.c12_0 c13_0 ib2.l10 i.l20 i.l30 if original==1 , base(2)

gen ind1 = runiform()

foreach a of numlist 0,1 {

gen ppr_pgd_`a'= exp(_b[0:_cons] + _b[0:1.a0]*(`a'==1) + _b[0:1.m0]*1.m0 + _b[0:1.c1_0]*1.c1_0 + _b[0:2.c1_0]*2.c1_0 /*

*/ + _b[0:2.c2_0]*2.c2_0 + _b[0:3.c2_0]*3.c2_0 + _b[0:4.c2_0]*4.c2_0 + _b[0:1.c3_0]*1.c3_0 + _b[0:1.c4_0]*1.c4_0 + _b[0:1.c5_0]*1.c5_0 /*

*/ + _b[0:1.c6_0]*1.c6_0 + _b[0:1.c7_0]*1.c7_0 + _b[0:2.c8_0]*2.c8_0 + _b[0:2.c9_0]*2.c9_0 +_b[0:3.c9_0]*3.c9_0 + _b[0:4.c9_0]*4.c9_0 /*

*/ + _b[0:c10_0]*c10_0 + _b[0:1.c11_0]*1.c11_0 + _b[0:2.c11_0]*2.c11_0 + _b[0:3.c11_0]*3.c11_0 + _b[0:2.c12_0]*2.c12_0 /*

*/ + _b[0:3.c12_0]*3.c12_0 + _b[0:4.c12_0]*4.c12_0 + _b[0:c13_0]*c13_0 /*

*/ + _b[0:0.l10]*0.l10 + _b[0:1.l10]*1.l10 + _b[0:3.l10]*3.l10 + _b[0:1.l20]*1.l20 + _b[0:2.l20]*2.l20 + _b[0:1.l30]*1.l30)

gen pfr_pgd_`a' = exp(_b[1:_cons] + _b[1:1.a0]*(`a'==1) + _b[1:1.m0]*1.m0 + _b[1:1.c1_0]*1.c1_0 + _b[1:2.c1_0]*2.c1_0 /*

*/ + _b[1:2.c2_0]*2.c2_0 + _b[1:3.c2_0]*3.c2_0 + _b[1:4.c2_0]*4.c2_0 + _b[1:1.c3_0]*1.c3_0 + _b[1:1.c4_0]*1.c4_0 + _b[1:1.c5_0]*1.c5_0 /*

*/ + _b[1:1.c6_0]*1.c6_0 + _b[1:1.c7_0]*1.c7_0 + _b[1:2.c8_0]*2.c8_0 + _b[1:2.c9_0]*2.c9_0 +_b[1:3.c9_0]*3.c9_0 + _b[1:4.c9_0]*4.c9_0 /*

*/ + _b[1:c10_0]*c10_0 + _b[1:1.c11_0]*1.c11_0 + _b[1:2.c11_0]*2.c11_0 + _b[1:3.c11_0]*3.c11_0 + _b[1:2.c12_0]*2.c12_0 /*

*/ + _b[1:3.c12_0]*3.c12_0 + _b[1:4.c12_0]*4.c12_0 + _b[1:c13_0]*c13_0 /*

*/ + _b[1:0.l10]*0.l10 + _b[1:1.l10]*1.l10 + _b[1:3.l10]*3.l10 + _b[1:1.l20]*1.l20 + _b[1:2.l20]*2.l20 + _b[1:1.l30]*1.l30)

gen pex_pgd_`a' = exp(_b[3:_cons] + _b[3:1.a0]*(`a'==1) + _b[3:1.m0]*1.m0 + _b[3:1.c1_0]*1.c1_0 + _b[3:2.c1_0]*2.c1_0 /*

*/ + _b[3:2.c2_0]*2.c2_0 + _b[3:3.c2_0]*3.c2_0 + _b[3:4.c2_0]*4.c2_0 + _b[3:1.c3_0]*1.c3_0 + _b[3:1.c4_0]*1.c4_0 + _b[3:1.c5_0]*1.c5_0 /*

*/ + _b[3:1.c6_0]*1.c6_0 + _b[3:1.c7_0]*1.c7_0 + _b[3:2.c8_0]*2.c8_0 + _b[3:2.c9_0]*2.c9_0 +_b[3:3.c9_0]*3.c9_0 + _b[3:4.c9_0]*4.c9_0 /*

*/ + _b[3:c10_0]*c10_0 + _b[3:1.c11_0]*1.c11_0 + _b[3:2.c11_0]*2.c11_0 + _b[3:3.c11_0]*3.c11_0 + _b[3:2.c12_0]*2.c12_0 /*

*/ + _b[3:3.c12_0]*3.c12_0 + _b[3:4.c12_0]*4.c12_0 + _b[3:c13_0]*c13_0 /*

*/ + _b[3:0.l10]*0.l10 + _b[3:1.l10]*1.l10 + _b[3:3.l10]*3.l10 + _b[3:1.l20]*1.l20 + _b[3:2.l20]*2.l20 + _b[3:1.l30]*1.l30)

gen prob_pr`a' = ppr_pgd_`a'/(1 + ppr_pgd_`a' + pfr_pgd_`a' + pex_pgd_`a')

gen prob_fr`a' = pfr_pgd_`a'/(1 + ppr_pgd_`a' + pfr_pgd_`a' + pex_pgd_`a')

gen prob_gd`a' = 1/(1 + ppr_pgd_`a' + pfr_pgd_`a' + pex_pgd_`a')

gen prob_ex`a' = pex_pgd_`a'/(1 + ppr_pgd_`a' + pfr_pgd_`a' + pex_pgd_`a')

gen l11_`a' = 0 if ind1< prob_pr`a'

replace l11_`a' = 1 if ind1>=prob_pr`a' & ind1<(prob_pr`a'+ prob_fr`a')

replace l11_`a' = 2 if ind1>=(prob_pr`a'+ prob_fr`a') & ind1< (prob_pr`a'+ prob_fr`a' + prob_gd`a')

replace l11_`a' = 3 if ind1>=(prob_pr`a'+ prob_fr`a' + prob_gd`a') & ind1!=.

}

*l21

mlogit l21 ib2.l11 i.a0 i.m0 i.c1_0 i.c2_0 i.c3_0 i.c4_0 i.c5_0 i.c6_0 i.c7_0 i.c8_0 i.c9_0 c10_0 i.c11_0 i.c12_0 c13_0 ib2.l10 i.l20 i.l30 if original==1

gen ind2 = runiform()

foreach a of numlist 0,1 {

gen pex_pnev_`a' = exp(_b[1:_cons] + _b[1:0.l11]*0.l11_`a' + _b[1:1.l11]*1.l11_`a' + _b[1:3.l11]*3.l11_`a' /*

*/ + _b[1:1.a0]*(`a'==1) + _b[1:1.m0]*1.m0 + _b[1:1.c1_0]*1.c1_0 + _b[1:2.c1_0]*2.c1_0 /*

*/ + _b[1:2.c2_0]*2.c2_0 + _b[1:3.c2_0]*3.c2_0 + _b[1:4.c2_0]*4.c2_0 + _b[1:1.c3_0]*1.c3_0 + _b[1:1.c4_0]*1.c4_0 + _b[1:1.c5_0]*1.c5_0 /*

*/ + _b[1:1.c6_0]*1.c6_0 + _b[1:1.c7_0]*1.c7_0 + _b[1:2.c8_0]*2.c8_0 + _b[1:2.c9_0]*2.c9_0 +_b[1:3.c9_0]*3.c9_0 + _b[1:4.c9_0]*4.c9_0 /*

*/ + _b[1:c10_0]*c10_0 + _b[1:1.c11_0]*1.c11_0 + _b[1:2.c11_0]*2.c11_0 + _b[1:3.c11_0]*3.c11_0 + _b[1:2.c12_0]*2.c12_0 /*

*/ + _b[1:3.c12_0]*3.c12_0 + _b[1:4.c12_0]*4.c12_0 + _b[1:c13_0]*c13_0 /*

*/ + _b[1:0.l10]*0.l10 + _b[1:1.l10]*1.l10 + _b[1:3.l10]*3.l10 + _b[1:1.l20]*1.l20 + _b[1:2.l20]*2.l20 + _b[1:1.l30]*1.l30)

gen pcur_pnev_`a' = exp(_b[2:_cons] + _b[2:0.l11]*0.l11_`a' + _b[2:1.l11]*1.l11_`a' + _b[2:3.l11]*3.l11_`a' /*

*/ + _b[2:1.a0]*(`a'==1) + _b[2:1.m0]*1.m0 + _b[2:1.c1_0]*1.c1_0 + _b[2:2.c1_0]*2.c1_0 /*

*/ + _b[2:2.c2_0]*2.c2_0 + _b[2:3.c2_0]*3.c2_0 + _b[2:4.c2_0]*4.c2_0 + _b[2:1.c3_0]*1.c3_0 + _b[2:1.c4_0]*1.c4_0 + _b[2:1.c5_0]*1.c5_0 /*

*/ + _b[2:1.c6_0]*1.c6_0 + _b[2:1.c7_0]*1.c7_0 + _b[2:2.c8_0]*2.c8_0 + _b[2:2.c9_0]*2.c9_0 +_b[2:3.c9_0]*3.c9_0 + _b[2:4.c9_0]*4.c9_0 /*

*/ + _b[2:c10_0]*c10_0 + _b[2:1.c11_0]*1.c11_0 + _b[2:2.c11_0]*2.c11_0 + _b[2:3.c11_0]*3.c11_0 + _b[2:2.c12_0]*2.c12_0 /*

*/ + _b[2:3.c12_0]*3.c12_0 + _b[2:4.c12_0]*4.c12_0 + _b[2:c13_0]*c13_0 /*

*/ + _b[2:0.l10]*0.l10 + _b[2:1.l10]*1.l10 + _b[2:3.l10]*3.l10 + _b[2:1.l20]*1.l20 + _b[2:2.l20]*2.l20 + _b[2:1.l30]*1.l30)

gen prob_never`a' = 1/(1+pex_pnev_`a' + pcur_pnev_`a')

gen prob_exsmk`a' = pex_pnev_`a'/(1+pex_pnev_`a' + pcur_pnev_`a')

gen prob_curr`a' = pcur_pnev_`a'/(1+pex_pnev_`a' + pcur_pnev_`a')

gen l21_`a' = 0 if ind2< prob_never`a'

replace l21_`a' = 1 if ind2>=prob_never`a' & ind2<(prob_never`a'+prob_exsmk`a')

replace l21_`a' = 2 if ind2>=(prob_never`a'+prob_exsmk`a') & ind2!=.

}

*l31

logit l31 i.l21 ib2.l11 i.a0 i.m0 i.c1_0 i.c2_0 i.c3_0 i.c4_0 i.c5_0 i.c6_0 i.c7_0 i.c8_0 i.c9_0 c10_0 i.c11_0 i.c12_0 c13_0 ib2.l10 i.l20 i.l30 if original==1

gen ind3 = runiform()

foreach a of numlist 0,1 {

gen l31_`a' = ind3 < 1/(1+exp(-(_b[_cons]+ _b[1.l21]*1.l21_`a' + _b[2.l21]*2.l21_`a' /*

*/ + _b[0.l11]*0.l11_`a' + _b[1.l11]*1.l11_`a' + _b[3.l11]*3.l11_`a' /*

*/ + _b[1.a0]*(`a'==1) + _b[1.m0]*1.m0 + _b[1.c1_0]*1.c1_0 + _b[2.c1_0]*2.c1_0 /*

*/ + _b[2.c2_0]*2.c2_0 + _b[3.c2_0]*3.c2_0 + _b[4.c2_0]*4.c2_0 + _b[1.c3_0]*1.c3_0 + _b[1.c4_0]*1.c4_0 + _b[1.c5_0]*1.c5_0 /*

*/ + _b[1.c6_0]*1.c6_0 + _b[1.c7_0]*1.c7_0 + _b[2.c8_0]*2.c8_0 + _b[2.c9_0]*2.c9_0 +_b[3.c9_0]*3.c9_0 + _b[4.c9_0]*4.c9_0 /*

*/ + _b[c10_0]*c10_0 + _b[1.c11_0]*1.c11_0 + _b[2.c11_0]*2.c11_0 + _b[3.c11_0]*3.c11_0 + _b[2.c12_0]*2.c12_0 + _b[3.c12_0]*3.c12_0 + _b[4.c12_0]*4.c12_0 + _b[c13_0]*c13_0/*

*/ + _b[0.l10]*0.l10 + _b[1.l10]*1.l10 + _b[3.l10]*3.l10 + _b[1.l20]*1.l20 + _b[2.l20]*2.l20 + _b[1.l30]*1.l30)))

}

*m1

logit m1 i.l31 i.l21 ib2.l11 i.a0 i.m0 i.c1_0 i.c2_0 i.c3_0 i.c4_0 i.c5_0 i.c6_0 i.c7_0 i.c8_0 i.c9_0 c10_0 i.c11_0 i.c12_0 c13_0 ib2.l10 i.l20 i.l30 if original==1

gen ind4 = runiform()

foreach a of numlist 0,1 {

gen m1_`a' = ind4 < 1/(1+exp(-(_b[_cons]+ _b[1.l31]*1.l31_`a' + _b[1.l21]*1.l21_`a' + _b[2.l21]*2.l21_`a' /*

*/ + _b[0.l11]*0.l11_`a' + _b[1.l11]*1.l11_`a' + _b[3.l11]*3.l11_`a' /*

*/ + _b[1.a0]*(`a'==1) + _b[1.m0]*1.m0 + _b[1.c1_0]*1.c1_0 + _b[2.c1_0]*2.c1_0 /*

*/ + _b[2.c2_0]*2.c2_0 + _b[3.c2_0]*3.c2_0 + _b[4.c2_0]*4.c2_0 + _b[1.c3_0]*1.c3_0 + _b[1.c4_0]*1.c4_0 + _b[1.c5_0]*1.c5_0 /*

*/ + _b[1.c6_0]*1.c6_0 + _b[1.c7_0]*1.c7_0 + _b[2.c8_0]*2.c8_0 + _b[2.c9_0]*2.c9_0 +_b[3.c9_0]*3.c9_0 + _b[4.c9_0]*4.c9_0 /*

*/ + _b[c10_0]*c10_0 + _b[1.c11_0]*1.c11_0 + _b[2.c11_0]*2.c11_0 + _b[3.c11_0]*3.c11_0 + _b[2.c12_0]*2.c12_0 + _b[3.c12_0]*3.c12_0 + _b[4.c12_0]*4.c12_0 + _b[c13_0]*c13_0/*

*/ + _b[0.l10]*0.l10 + _b[1.l10]*1.l10 + _b[3.l10]*3.l10 + _b[1.l20]*1.l20 + _b[2.l20]*2.l20 + _b[1.l30]*1.l30)))

}

* l12

mlogit l12 i.a1 i.m1 ib2.l11 i.l21 i.l31 i.a0 i.m0 i.c1_0 i.c2_0 i.c3_0 i.c4_0 i.c5_0 i.c6_0 i.c7_0 i.c8_0 i.c9_0 c10_0 i.c11_0 i.c12_0 c13_0 /*

*/ ib2.l10 i.l20 i.l30 if original==1 ,base(2)

gen ind5 = runiform()

foreach a0 of numlist 0 1 {

foreach a1 of numlist 0,1 {

gen ppr_pgd2_`a0'`a1' = exp(_b[0:_cons] + _b[0:1.a1]*(`a1'==1) + _b[0:1.m1]*1.m1_`a0' + _b[0:0.l11]*0.l11_`a0' + _b[0:1.l11]*1.l11_`a0' /*

*/ + _b[0:3.l11]*3.l11_`a0' + _b[0:1.l21]*1.l21_`a0' + _b[0:2.l21]*2.l21_`a0' + _b[0:1.l31]*1.l31_`a0' + _b[0:1.a0]*(`a0'==1) /*

*/ + _b[0:1.m0]*1.m0 + _b[0:1.c1_0]*1.c1_0 + _b[0:2.c1_0]*2.c1_0 /*

*/ + _b[0:2.c2_0]*2.c2_0 + _b[0:3.c2_0]*3.c2_0 + _b[0:4.c2_0]*4.c2_0 + _b[0:1.c3_0]*1.c3_0 + _b[0:1.c4_0]*1.c4_0 + _b[0:1.c5_0]*1.c5_0 /*

*/ + _b[0:1.c6_0]*1.c6_0 + _b[0:1.c7_0]*1.c7_0 + _b[0:2.c8_0]*2.c8_0 + _b[0:2.c9_0]*2.c9_0 +_b[0:3.c9_0]*3.c9_0 + _b[0:4.c9_0]*4.c9_0 /*

*/ + _b[0:c10_0]*c10_0 + _b[0:1.c11_0]*1.c11_0 + _b[0:2.c11_0]*2.c11_0 + _b[0:3.c11_0]*3.c11_0 + _b[0:2.c12_0]*2.c12_0 + _b[0:c13_0]*c13_0 /*

*/ + _b[0:3.c12_0]*3.c12_0 + _b[0:4.c12_0]*4.c12_0 /*

*/ + _b[0:0.l10]*0.l10 + _b[0:1.l10]*1.l10 + _b[0:3.l10]*3.l10 + _b[0:1.l20]*1.l20 + _b[0:2.l20]*2.l20 + _b[0:1.l30]*1.l30)

gen pfr_pgd2_`a0'`a1' = exp(_b[1:_cons] + _b[1:1.a1]*(`a1'==1) + _b[1:1.m1]*1.m1_`a0' + _b[1:0.l11]*0.l11_`a0' + _b[1:1.l11]*1.l11_`a0' /*

*/ + _b[1:3.l11]*3.l11_`a0' + _b[1:1.l21]*1.l21_`a0' + _b[1:2.l21]*2.l21_`a0' + _b[1:1.l31]*1.l31_`a0' + _b[1:1.a0]*(`a0'==1) /*

*/ + _b[1:1.m0]*1.m0 + _b[1:1.c1_0]*1.c1_0 + _b[1:2.c1_0]*2.c1_0 /*

*/ + _b[1:2.c2_0]*2.c2_0 + _b[1:3.c2_0]*3.c2_0 + _b[1:4.c2_0]*4.c2_0 + _b[1:1.c3_0]*1.c3_0 + _b[1:1.c4_0]*1.c4_0 + _b[1:1.c5_0]*1.c5_0 /*

*/ + _b[1:1.c6_0]*1.c6_0 + _b[1:1.c7_0]*1.c7_0 + _b[1:2.c8_0]*2.c8_0 + _b[1:2.c9_0]*2.c9_0 +_b[1:3.c9_0]*3.c9_0 + _b[1:4.c9_0]*4.c9_0 /*

*/ + _b[1:c10_0]*c10_0 + _b[1:1.c11_0]*1.c11_0 + _b[1:2.c11_0]*2.c11_0 + _b[1:3.c11_0]*3.c11_0 + _b[1:2.c12_0]*2.c12_0 /*

*/ + _b[1:3.c12_0]*3.c12_0 + _b[1:4.c12_0]*4.c12_0 + _b[1:c13_0]*c13_0 /*

*/ + _b[1:0.l10]*0.l10 + _b[1:1.l10]*1.l10 + _b[1:3.l10]*3.l10 + _b[1:1.l20]*1.l20 + _b[1:2.l20]*2.l20 + _b[1:1.l30]*1.l30)

gen pex_pgd2_`a0'`a1' = exp(_b[3:_cons] + _b[3:1.a1]*(`a1'==1) + _b[3:1.m1]*1.m1_`a0' + _b[3:0.l11]*0.l11_`a0' + _b[3:1.l11]*1.l11_`a0' /*

*/ + _b[3:3.l11]*3.l11_`a0' + _b[3:1.l21]*1.l21_`a0' + _b[3:2.l21]*2.l21_`a0' + _b[3:1.l31]*1.l31_`a0' + _b[3:1.a0]*(`a0'==1) /*

*/ + _b[3:1.m0]*1.m0 + _b[3:1.c1_0]*1.c1_0 + _b[3:2.c1_0]*2.c1_0 /*

*/ + _b[3:2.c2_0]*2.c2_0 + _b[3:3.c2_0]*3.c2_0 + _b[3:4.c2_0]*4.c2_0 + _b[3:1.c3_0]*1.c3_0 + _b[3:1.c4_0]*1.c4_0 + _b[3:1.c5_0]*1.c5_0 /*

*/ + _b[3:1.c6_0]*1.c6_0 + _b[3:1.c7_0]*1.c7_0 + _b[3:2.c8_0]*2.c8_0 + _b[3:2.c9_0]*2.c9_0 +_b[3:3.c9_0]*3.c9_0 + _b[3:4.c9_0]*4.c9_0 /*

*/ + _b[3:c10_0]*c10_0 + _b[3:1.c11_0]*1.c11_0 + _b[3:2.c11_0]*2.c11_0 + _b[3:3.c11_0]*3.c11_0 + _b[3:2.c12_0]*2.c12_0 /*

*/ + _b[3:3.c12_0]*3.c12_0 + _b[3:4.c12_0]*4.c12_0 + _b[3:c13_0]*c13_0 /*

*/ + _b[3:0.l10]*0.l10 + _b[3:1.l10]*1.l10 + _b[3:3.l10]*3.l10 + _b[3:1.l20]*1.l20 + _b[3:2.l20]*2.l20 + _b[3:1.l30]*1.l30)

gen prob_pr2`a0'`a1' = ppr_pgd2_`a0'`a1'/(1 + ppr_pgd2_`a0'`a1' + pfr_pgd2_`a0'`a1' + pex_pgd2_`a0'`a1')

gen prob_fr2`a0'`a1' = pfr_pgd2_`a0'`a1'/(1 + ppr_pgd2_`a0'`a1' + pfr_pgd2_`a0'`a1' + pex_pgd2_`a0'`a1')

gen prob_gd2`a0'`a1' = 1/(1 + ppr_pgd2_`a0'`a1' + pfr_pgd2_`a0'`a1' + pex_pgd2_`a0'`a1' )

gen prob_ex2`a0'`a1' = pex_pgd2_`a0'`a1' /(1 + ppr_pgd2_`a0'`a1' + pfr_pgd2_`a0'`a1' + pex_pgd2_`a0'`a1')

gen l12_`a0'`a1' = 0 if ind5< prob_pr2`a0'`a1'

replace l12_`a0'`a1' = 1 if ind5>=prob_pr2`a0'`a1' & ind5<(prob_pr2`a0'`a1' + prob_fr2`a0'`a1' )

replace l12_`a0'`a1' = 2 if ind5>=(prob_pr2`a0'`a1' + prob_fr2`a0'`a1' ) & ind5< (prob_pr2`a0'`a1' + prob_fr2`a0'`a1' + prob_gd2`a0'`a1' )

replace l12_`a0'`a1' = 3 if ind5>=(prob_pr2`a0'`a1' + prob_fr2`a0'`a1' + prob_gd2`a0'`a1' ) & ind5!=.

}

}

*l22

mlogit l22 ib2.l12 i.a1 i.m1 ib2.l11 i.l21 i.l31 i.a0 i.m0 i.c1_0 i.c2_0 i.c3_0 i.c4_0 i.c5_0 i.c6_0 i.c7_0 i.c8_0 i.c9_0 c10_0 i.c11_0 i.c12_0 c13_0 /*

*/ ib2.l10 i.l20 i.l30 if original==1

gen ind6 = runiform()

foreach a0 of numlist 0,1 {

foreach a1 of numlist 0,1 {

gen pex_pnev_2`a0'`a1' = exp(_b[1:_cons] + _b[1:0.l12]*0.l12_`a0'`a1' + _b[1:1.l12]*1.l12_`a0'`a1' + _b[1:3.l12]*3.l12_`a0'`a1' /*

*/ + _b[1:1.a1]*(`a1'==1) + _b[1:1.m1]*1.m1_`a0' + _b[1:0.l11]*0.l11_`a0' + _b[1:1.l11]*1.l11_`a0' /*

*/ + _b[1:3.l11]*3.l11_`a0' + _b[1:1.l21]*1.l21_`a0' + _b[1:2.l21]*2.l21_`a0' + _b[1:1.l31]*1.l31_`a0' /*

*/ + _b[1:1.a0]*(`a0'==1) + _b[1:1.m0]*1.m0 + _b[1:1.c1_0]*1.c1_0 + _b[1:2.c1_0]*2.c1_0 /*

*/ + _b[1:2.c2_0]*2.c2_0 + _b[1:3.c2_0]*3.c2_0 + _b[1:4.c2_0]*4.c2_0 + _b[1:1.c3_0]*1.c3_0 + _b[1:1.c4_0]*1.c4_0 + _b[1:1.c5_0]*1.c5_0 /*

*/ + _b[1:1.c6_0]*1.c6_0 + _b[1:1.c7_0]*1.c7_0 + _b[1:2.c8_0]*2.c8_0 + _b[1:2.c9_0]*2.c9_0 +_b[1:3.c9_0]*3.c9_0 + _b[1:4.c9_0]*4.c9_0 /*

*/ + _b[1:c10_0]*c10_0 + _b[1:1.c11_0]*1.c11_0 + _b[1:2.c11_0]*2.c11_0 + _b[1:3.c11_0]*3.c11_0 + _b[1:2.c12_0]*2.c12_0 /*

*/ + _b[1:3.c12_0]*3.c12_0 + _b[1:4.c12_0]*4.c12_0 + _b[1:c13_0]*c13_0 /*

*/ + _b[1:0.l10]*0.l10 + _b[1:1.l10]*1.l10 + _b[1:3.l10]*3.l10 + _b[1:1.l20]*1.l20 + _b[1:2.l20]*2.l20 + _b[1:1.l30]*1.l30)

gen pcur_pnev_2`a0'`a1' = exp(_b[2:_cons] + _b[2:0.l12]*0.l12_`a0'`a1' + _b[2:1.l12]*1.l12_`a0'`a1' + _b[2:3.l12]*3.l12_`a0'`a1' /*

*/ + _b[2:1.a1]*(`a1'==1) + _b[2:1.m1]*1.m1_`a0' + _b[2:0.l11]*0.l11_`a0' + _b[2:1.l11]*1.l11_`a0' /*

*/ + _b[2:3.l11]*3.l11_`a0' + _b[2:1.l21]*1.l21_`a0' + _b[2:2.l21]*2.l21_`a0' + _b[2:1.l31]*1.l31_`a0' /*

*/ + _b[2:1.a0]*(`a0'==1) + _b[2:1.m0]*1.m0 + _b[2:1.c1_0]*1.c1_0 + _b[2:2.c1_0]*2.c1_0 /*

*/ + _b[2:2.c2_0]*2.c2_0 + _b[2:3.c2_0]*3.c2_0 + _b[2:4.c2_0]*4.c2_0 + _b[2:1.c3_0]*1.c3_0 + _b[2:1.c4_0]*1.c4_0 + _b[2:1.c5_0]*1.c5_0 /*

*/ + _b[2:1.c6_0]*1.c6_0 + _b[2:1.c7_0]*1.c7_0 + _b[2:2.c8_0]*2.c8_0 + _b[2:2.c9_0]*2.c9_0 +_b[2:3.c9_0]*3.c9_0 + _b[2:4.c9_0]*4.c9_0 /*

*/ + _b[2:c10_0]*c10_0 + _b[2:1.c11_0]*1.c11_0 + _b[2:2.c11_0]*2.c11_0 + _b[2:3.c11_0]*3.c11_0 + _b[2:2.c12_0]*2.c12_0 /*

*/ + _b[2:3.c12_0]*3.c12_0 + _b[2:4.c12_0]*4.c12_0 + _b[2:c13_0]*c13_0/*

*/ + _b[2:0.l10]*0.l10 + _b[2:1.l10]*1.l10 + _b[2:3.l10]*3.l10 + _b[2:1.l20]*1.l20 + _b[2:2.l20]*2.l20 + _b[2:1.l30]*1.l30)

gen prob_never2`a0'`a1' = 1/(1+pex_pnev_2`a0'`a1' + pcur_pnev_2`a0'`a1')

gen prob_exsmk2`a0'`a1' = pex_pnev_2`a0'`a1'/(1+pex_pnev_2`a0'`a1' + pcur_pnev_2`a0'`a1' )

gen prob_curr2`a0'`a1' = pcur_pnev_2`a0'`a1'/(1+pex_pnev_2`a0'`a1' + pcur_pnev_2`a0'`a1')

gen l22_`a0'`a1' = 0 if ind6< prob_never2`a0'`a1'

replace l22_`a0'`a1' = 1 if ind6>=prob_never2`a0'`a1' & ind6<(prob_never2`a0'`a1' + prob_exsmk2`a0'`a1' )

replace l22_`a0'`a1' = 2 if ind6>=(prob_never2`a0'`a1' + prob_exsmk2`a0'`a1' ) & ind6!=.

}

}

*l32

logit l32 i.l22 ib2.l12 i.a1 i.m1 ib2.l11 i.l21 i.l31 i.a0 i.m0 i.c1_0 i.c2_0 i.c3_0 i.c4_0 i.c5_0 i.c6_0 i.c7_0 i.c8_0 i.c9_0 c10_0 i.c11_0 i.c12_0 c13_0 /*

*/ ib2.l10 i.l20 i.l30 if original==1

gen ind7 = runiform()

foreach a0 of numlist 0, 1 {

foreach a1 of numlist 0,1 {

gen l32_`a0'`a1' = ind7< 1/(1+exp(-(_b[_cons]+ _b[1.l22]*1.l22_`a0'`a1' + _b[2.l22]*2.l22_`a0'`a1' /*

*/ + _b[0.l12]*0.l12_`a0'`a1' + _b[1.l12]*1.l12_`a0'`a1' + _b[3.l12]*3.l12_`a0'`a1' /*

*/ + _b[1.a1]*(`a1'==1) + _b[1.m1]*1.m1_`a0' + _b[0.l11]*0.l11_`a0' + _b[1.l11]*1.l11_`a0' /*

*/ + _b[3.l11]*3.l11_`a0' + _b[1.l21]*1.l21_`a0' + _b[2.l21]*2.l21_`a0' + _b[1.l31]*1.l31_`a0' /*

*/ + _b[1.a0]*(`a0'==1) + _b[1.m0]*1.m0 + _b[1.c1_0]*1.c1_0 + _b[2.c1_0]*2.c1_0 /*

*/ + _b[2.c2_0]*2.c2_0 + _b[3.c2_0]*3.c2_0 + _b[4.c2_0]*4.c2_0 + _b[1.c3_0]*1.c3_0 + _b[1.c4_0]*1.c4_0 + _b[1.c5_0]*1.c5_0 /*

*/ + _b[1.c6_0]*1.c6_0 + _b[1.c7_0]*1.c7_0 + _b[2.c8_0]*2.c8_0 + _b[2.c9_0]*2.c9_0 +_b[3.c9_0]*3.c9_0 + _b[4.c9_0]*4.c9_0 /*

*/ + _b[c10_0]*c10_0 + _b[1.c11_0]*1.c11_0 + _b[2.c11_0]*2.c11_0 + _b[3.c11_0]*3.c11_0 + _b[2.c12_0]*2.c12_0 + _b[3.c12_0]*3.c12_0 + _b[4.c12_0]*4.c12_0 + _b[c13_0]*c13_0 /*

*/ + _b[0.l10]*0.l10 + _b[1.l10]*1.l10 + _b[3.l10]*3.l10 + _b[1.l20]*1.l20 + _b[2.l20]*2.l20 + _b[1.l30]*1.l30)))

}

}

*m2

logit m2 i.l32 i.l22 ib2.l12 i.a1 i.m1 ib2.l11 i.l21 i.l31 i.a0 i.m0 i.c1_0 i.c2_0 i.c3_0 i.c4_0 i.c5_0 i.c6_0 i.c7_0 i.c8_0 i.c9_0 c10_0 /*

*/ i.c11_0 i.c12_0 c13_0 ib2.l10 i.l20 i.l30 if original==1

gen ind8 = runiform()

foreach a0 of numlist 0,1 {

foreach a1 of numlist 0, 1 {

gen m2_`a0'`a1' = ind8< 1/(1+exp(-(_b[_cons]+ _b[1.l32]*1.l32_`a0'`a1' + _b[1.l22]*1.l22_`a0'`a1' + _b[2.l22]*2.l22_`a0'`a1' /*

*/ + _b[0.l12]*0.l12_`a0'`a1' + _b[1.l12]*1.l12_`a0'`a1' + _b[3.l12]*3.l12_`a0'`a1' + _b[1.a1]*(`a1'==1) + _b[1.m1]*1.m1_`a0' + /*

*/ _b[0.l11]*0.l11_`a0' + _b[1.l11]*1.l11_`a0' + _b[3.l11]*3.l11_`a0' + /*

*/ _b[1.l21]*1.l21_`a0' + _b[2.l21]*2.l21_`a0' + _b[1.l31]*1.l31_`a0' + _b[1.a0]*(`a0'==1) + _b[1.m0]*1.m0 + _b[1.c1_0]*1.c1_0 + _b[2.c1_0]*2.c1_0 /*

*/ + _b[2.c2_0]*2.c2_0 + _b[3.c2_0]*3.c2_0 + _b[4.c2_0]*4.c2_0 + _b[1.c3_0]*1.c3_0 + _b[1.c4_0]*1.c4_0 + _b[1.c5_0]*1.c5_0 /*

*/ + _b[1.c6_0]*1.c6_0 + _b[1.c7_0]*1.c7_0 + _b[2.c8_0]*2.c8_0 + _b[2.c9_0]*2.c9_0 +_b[3.c9_0]*3.c9_0 + _b[4.c9_0]*4.c9_0 /*

*/ + _b[c10_0]*c10_0 + _b[1.c11_0]*1.c11_0 + _b[2.c11_0]*2.c11_0 + _b[3.c11_0]*3.c11_0 + _b[2.c12_0]*2.c12_0 + _b[3.c12_0]*3.c12_0 + _b[4.c12_0]*4.c12_0 + _b[c13_0]*c13_0 /*

*/ + _b[0.l10]*0.l10 + _b[1.l10]*1.l10 + _b[3.l10]*3.l10 + _b[1.l20]*1.l20 + _b[2.l20]*2.l20 + _b[1.l30]*1.l30)))

}

}

***********************

****PART 2b & 2c & 2d

***********************

*permute predicted mediators

scalar N=_N

foreach a0 of numlist 0,1 {

sort ncdsid

gen new`a0'=(runiform(1,N))

sort new`a0'

gen rank`a0'=_n

sort ncdsid

gen G_M1_`a0'=m1_`a0'[rank`a0']

}

foreach a0 of numlist 0,1 {

foreach a1 of numlist 0,1 {

sort ncdsid

gen new`a0'`a1'=(runiform(1,N))

sort new`a0'`a1'

gen rank`a0'`a1' =_n

sort ncdsid

gen G_M2_`a0'`a1' = m2_`a0'`a1'[rank`a0'`a1']

}

}

***********************

****PART 3

***********************

* l12

mlogit l12 i.a1 i.m1 ib2.l11 i.l21 i.l31 i.a0 i.m0 i.c1_0 i.c2_0 i.c3_0 i.c4_0 i.c5_0 i.c6_0 i.c7_0 i.c8_0 i.c9_0 c10_0 i.c11_0 i.c12_0 c13_0 /*

*/ ib2.l10 i.l20 i.l30 if original==1 ,base(2)

foreach a0 of numlist 0,1 { /*index for a0 */

foreach a1 of numlist 0,1 { /*index for a1 */

foreach m of numlist 0,1 { /*index for m */

gen ppr_pgd2_`a0'`a1'_m1_`m' = exp(_b[0:_cons] + _b[0:1.a1]*(`a1'==1) + _b[0:1.m1]*1.G_M1_`m' + _b[0:0.l11]*0.l11_`a0' + _b[0:1.l11]*1.l11_`a0' /*

*/ + _b[0:3.l11]*3.l11_`a0' + _b[0:1.l21]*1.l21_`a0' + _b[0:2.l21]*2.l21_`a0' + _b[0:1.l31]*1.l31_`a0' + _b[0:1.a0]*(`a0'==1) /*

*/ + _b[0:1.m0]*1.m0 + _b[0:1.c1_0]*1.c1_0 + _b[0:2.c1_0]*2.c1_0 /*

*/ + _b[0:2.c2_0]*2.c2_0 + _b[0:3.c2_0]*3.c2_0 + _b[0:4.c2_0]*4.c2_0 + _b[0:1.c3_0]*1.c3_0 + _b[0:1.c4_0]*1.c4_0 + _b[0:1.c5_0]*1.c5_0 /*

*/ + _b[0:1.c6_0]*1.c6_0 + _b[0:1.c7_0]*1.c7_0 + _b[0:2.c8_0]*2.c8_0 + _b[0:2.c9_0]*2.c9_0 +_b[0:3.c9_0]*3.c9_0 + _b[0:4.c9_0]*4.c9_0 /*

*/ + _b[0:c10_0]*c10_0 + _b[0:1.c11_0]*1.c11_0 + _b[0:2.c11_0]*2.c11_0 + _b[0:3.c11_0]*3.c11_0 + _b[0:2.c12_0]*2.c12_0 /*

*/ + _b[0:3.c12_0]*3.c12_0 + _b[0:4.c12_0]*4.c12_0 + _b[0:c13_0]*c13_0 /*

*/ + _b[0:0.l10]*0.l10 + _b[0:1.l10]*1.l10 + _b[0:3.l10]*3.l10 + _b[0:1.l20]*1.l20 + _b[0:2.l20]*2.l20 + _b[0:1.l30]*1.l30)

gen pfr_pgd2_`a0'`a1'_m1_`m' = exp(_b[1:_cons] + _b[1:1.a1]*(`a1'==1) + _b[1:1.m1]*1.G_M1_`m' + _b[1:0.l11]*0.l11_`a0' + _b[1:1.l11]*1.l11_`a0' /*

*/ + _b[1:3.l11]*3.l11_`a0' + _b[1:1.l21]*1.l21_`a0' + _b[1:2.l21]*2.l21_`a0' + _b[1:1.l31]*1.l31_`a0' + _b[1:1.a0]*(`a0'==1) /*

*/ + _b[1:1.m0]*1.m0 + _b[1:1.c1_0]*1.c1_0 + _b[1:2.c1_0]*2.c1_0 /*

*/ + _b[1:2.c2_0]*2.c2_0 + _b[1:3.c2_0]*3.c2_0 + _b[1:4.c2_0]*4.c2_0 + _b[1:1.c3_0]*1.c3_0 + _b[1:1.c4_0]*1.c4_0 + _b[1:1.c5_0]*1.c5_0 /*

*/ + _b[1:1.c6_0]*1.c6_0 + _b[1:1.c7_0]*1.c7_0 + _b[1:2.c8_0]*2.c8_0 + _b[1:2.c9_0]*2.c9_0 +_b[1:3.c9_0]*3.c9_0 + _b[1:4.c9_0]*4.c9_0 /*

*/ + _b[1:c10_0]*c10_0 + _b[1:1.c11_0]*1.c11_0 + _b[1:2.c11_0]*2.c11_0 + _b[1:3.c11_0]*3.c11_0 + _b[1:2.c12_0]*2.c12_0 /*

*/ + _b[1:3.c12_0]*3.c12_0 + _b[1:4.c12_0]*4.c12_0 + _b[1:c13_0]*c13_0 /*

*/ + _b[1:0.l10]*0.l10 + _b[1:1.l10]*1.l10 + _b[1:3.l10]*3.l10 + _b[1:1.l20]*1.l20 + _b[1:2.l20]*2.l20 + _b[1:1.l30]*1.l30)

gen pex_pgd2_`a0'`a1'_m1_`m' = exp(_b[3:_cons] + _b[3:1.a1]*(`a1'==1) + _b[3:1.m1]*1.G_M1_`m' + _b[3:0.l11]*0.l11_`a0' + _b[3:1.l11]*1.l11_`a0' /*

*/ + _b[3:3.l11]*3.l11_`a0' + _b[3:1.l21]*1.l21_`a0' + _b[3:2.l21]*2.l21_`a0' + _b[3:1.l31]*1.l31_`a0' + _b[3:1.a0]*(`a0'==1) /*

*/ + _b[3:1.m0]*1.m0 + _b[3:1.c1_0]*1.c1_0 + _b[3:2.c1_0]*2.c1_0 /*

*/ + _b[3:2.c2_0]*2.c2_0 + _b[3:3.c2_0]*3.c2_0 + _b[3:4.c2_0]*4.c2_0 + _b[3:1.c3_0]*1.c3_0 + _b[3:1.c4_0]*1.c4_0 + _b[3:1.c5_0]*1.c5_0 /*

*/ + _b[3:1.c6_0]*1.c6_0 + _b[3:1.c7_0]*1.c7_0 + _b[3:2.c8_0]*2.c8_0 + _b[3:2.c9_0]*2.c9_0 +_b[3:3.c9_0]*3.c9_0 + _b[3:4.c9_0]*4.c9_0 /*

*/ + _b[3:c10_0]*c10_0 + _b[3:1.c11_0]*1.c11_0 + _b[3:2.c11_0]*2.c11_0 + _b[3:3.c11_0]*3.c11_0 + _b[3:2.c12_0]*2.c12_0 /*

*/ + _b[3:3.c12_0]*3.c12_0 + _b[3:4.c12_0]*4.c12_0 + _b[3:c13_0]*c13_0 /*

*/ + _b[3:0.l10]*0.l10 + _b[3:1.l10]*1.l10 + _b[3:3.l10]*3.l10 + _b[3:1.l20]*1.l20 + _b[3:2.l20]*2.l20 + _b[3:1.l30]*1.l30)

gen prob_pr2`a0'`a1'_m1_`m' = ppr_pgd2_`a0'`a1'_m1_`m'/(1 + ppr_pgd2_`a0'`a1'_m1_`m' + pfr_pgd2_`a0'`a1'_m1_`m' + pex_pgd2_`a0'`a1'_m1_`m')

gen prob_fr2`a0'`a1'_m1_`m' = pfr_pgd2_`a0'`a1'_m1_`m'/(1 + ppr_pgd2_`a0'`a1'_m1_`m' + pfr_pgd2_`a0'`a1'_m1_`m' + pex_pgd2_`a0'`a1'_m1_`m')

gen prob_gd2`a0'`a1'_m1_`m' = 1/(1 + ppr_pgd2_`a0'`a1'_m1_`m' + pfr_pgd2_`a0'`a1'_m1_`m' + pex_pgd2_`a0'`a1'_m1_`m')

gen prob_ex2`a0'`a1'_m1_`m' = pex_pgd2_`a0'`a1'_m1_`m'/(1 + ppr_pgd2_`a0'`a1'_m1_`m' + pfr_pgd2_`a0'`a1'_m1_`m' + pex_pgd2_`a0'`a1'_m1_`m')

gen l12_`a0'`a1'_m1_`m' = 0 if ind5< prob_pr2`a0'`a1'_m1_`m'

replace l12_`a0'`a1'_m1_`m' = 1 if ind5>=prob_pr2`a0'`a1'_m1_`m' & ind5<(prob_pr2`a0'`a1'_m1_`m' + prob_fr2`a0'`a1'_m1_`m')

replace l12_`a0'`a1'_m1_`m' = 2 if ind5>=(prob_pr2`a0'`a1'_m1_`m' + prob_fr2`a0'`a1'_m1_`m') & ind5< (prob_pr2`a0'`a1'_m1_`m' + prob_fr2`a0'`a1'_m1_`m' + prob_gd2`a0'`a1'_m1_`m')

replace l12_`a0'`a1'_m1_`m' = 3 if ind5>=(prob_pr2`a0'`a1'_m1_`m' + prob_fr2`a0'`a1'_m1_`m' + prob_gd2`a0'`a1'_m1_`m') & ind5!=.

}

}

}

*l22

mlogit l22 ib2.l12 i.a1 i.m1 ib2.l11 i.l21 i.l31 i.a0 i.m0 i.c1_0 i.c2_0 i.c3_0 i.c4_0 i.c5_0 i.c6_0 i.c7_0 i.c8_0 i.c9_0 c10_0 i.c11_0 i.c12_0 c13_0 /*

*/ ib2.l10 i.l20 i.l30 if original==1

foreach a0 of numlist 0,1 { /*index for a0 */

foreach a1 of numlist 0,1 { /*index for a1 */

foreach m of numlist 0,1 { /*index for m */

gen pex_pnev_2`a0'`a1'_m1_`m' = exp(_b[1:_cons] + _b[1:0.l12]*0.l12_`a0'`a1'_m1_`m' + _b[1:1.l12]*1.l12_`a0'`a1'_m1_`m' + _b[1:3.l12]*3.l12_`a0'`a1'_m1_`m' /*

*/ + _b[1:1.a1]*(`a1'==1) + _b[1:1.m1]*1.G_M1_`m' + _b[1:0.l11]*0.l11_`a0' + _b[1:1.l11]*1.l11_`a0' /*

*/ + _b[1:3.l11]*3.l11_`a0' + _b[1:1.l21]*1.l21_`a0' + _b[1:2.l21]*2.l21_`a0' + _b[1:1.l31]*1.l31_`a0' /*

*/ + _b[1:1.a0]*(`a0'==1) + _b[1:1.m0]*1.m0 + _b[1:1.c1_0]*1.c1_0 + _b[1:2.c1_0]*2.c1_0 /*

*/ + _b[1:2.c2_0]*2.c2_0 + _b[1:3.c2_0]*3.c2_0 + _b[1:4.c2_0]*4.c2_0 + _b[1:1.c3_0]*1.c3_0 + _b[1:1.c4_0]*1.c4_0 + _b[1:1.c5_0]*1.c5_0 /*

*/ + _b[1:1.c6_0]*1.c6_0 + _b[1:1.c7_0]*1.c7_0 + _b[1:2.c8_0]*2.c8_0 + _b[1:2.c9_0]*2.c9_0 +_b[1:3.c9_0]*3.c9_0 + _b[1:4.c9_0]*4.c9_0 /*

*/ + _b[1:c10_0]*c10_0 + _b[1:1.c11_0]*1.c11_0 + _b[1:2.c11_0]*2.c11_0 + _b[1:3.c11_0]*3.c11_0 + _b[1:2.c12_0]*2.c12_0 /*

*/ + _b[1:3.c12_0]*3.c12_0 + _b[1:4.c12_0]*4.c12_0 + _b[1:c13_0]*c13_0/*

*/ + _b[1:0.l10]*0.l10 + _b[1:1.l10]*1.l10 + _b[1:3.l10]*3.l10 + _b[1:1.l20]*1.l20 + _b[1:2.l20]*2.l20 + _b[1:1.l30]*1.l30)

gen pcur_pnev_2`a0'`a1'_m1_`m' = exp(_b[2:_cons] + _b[2:0.l12]*0.l12_`a0'`a1'_m1_`m' + _b[2:1.l12]*1.l12_`a0'`a1'_m1_`m' + _b[2:3.l12]*3.l12_`a0'`a1'_m1_`m' /*

*/ + _b[2:1.a1]*(`a1'==1) + _b[2:1.m1]*1.G_M1_`m' + _b[2:0.l11]*0.l11_`a0' + _b[2:1.l11]*1.l11_`a0' /*

*/ + _b[2:3.l11]*3.l11_`a0' + _b[2:1.l21]*1.l21_`a0' + _b[2:2.l21]*2.l21_`a0' + _b[2:1.l31]*1.l31_`a0' /*

*/ + _b[2:1.a0]*(`a0'==1) + _b[2:1.m0]*1.m0 + _b[2:1.c1_0]*1.c1_0 + _b[2:2.c1_0]*2.c1_0 /*

*/ + _b[2:2.c2_0]*2.c2_0 + _b[2:3.c2_0]*3.c2_0 + _b[2:4.c2_0]*4.c2_0 + _b[2:1.c3_0]*1.c3_0 + _b[2:1.c4_0]*1.c4_0 + _b[2:1.c5_0]*1.c5_0 /*

*/ + _b[2:1.c6_0]*1.c6_0 + _b[2:1.c7_0]*1.c7_0 + _b[2:2.c8_0]*2.c8_0 + _b[2:2.c9_0]*2.c9_0 +_b[2:3.c9_0]*3.c9_0 + _b[2:4.c9_0]*4.c9_0 /*

*/ + _b[2:c10_0]*c10_0 + _b[2:1.c11_0]*1.c11_0 + _b[2:2.c11_0]*2.c11_0 + _b[2:3.c11_0]*3.c11_0 + _b[2:2.c12_0]*2.c12_0 /*

*/ + _b[2:3.c12_0]*3.c12_0 + _b[2:4.c12_0]*4.c12_0 + _b[2:c13_0]*c13_0 /*

*/ + _b[2:0.l10]*0.l10 + _b[2:1.l10]*1.l10 + _b[2:3.l10]*3.l10 + _b[2:1.l20]*1.l20 + _b[2:2.l20]*2.l20 + _b[2:1.l30]*1.l30)

gen prob_never2`a0'`a1'_m1_`m' = 1/(1+pex_pnev_2`a0'`a1'_m1_`m' + pcur_pnev_2`a0'`a1'_m1_`m')

gen prob_exsmk2`a0'`a1'_m1_`m' = pex_pnev_2`a0'`a1'_m1_`m'/(1+pex_pnev_2`a0'`a1'_m1_`m' + pcur_pnev_2`a0'`a1'_m1_`m')

gen prob_curr2`a0'`a1'_m1_`m' = pcur_pnev_2`a0'`a1'_m1_`m'/(1+pex_pnev_2`a0'`a1'_m1_`m' + pcur_pnev_2`a0'`a1'_m1_`m')

gen l22_`a0'`a1'_m1_`m' = 0 if ind6< prob_never2`a0'`a1'_m1_`m'

replace l22_`a0'`a1'_m1_`m' = 1 if ind6>=prob_never2`a0'`a1'_m1_`m' & ind6<(prob_never2`a0'`a1'_m1_`m'+prob_exsmk2`a0'`a1'_m1_`m')

replace l22_`a0'`a1'_m1_`m' = 2 if ind6>=(prob_never2`a0'`a1'_m1_`m'+prob_exsmk2`a0'`a1'_m1_`m') & ind6!=.

}

}

}

*l32

logit l32 i.l22 ib2.l12 i.a1 i.m1 ib2.l11 i.l21 i.l31 i.a0 i.m0 i.c1_0 i.c2_0 i.c3_0 i.c4_0 i.c5_0 i.c6_0 i.c7_0 i.c8_0 i.c9_0 c10_0 i.c11_0 i.c12_0 c13_0 /*

*/ ib2.l10 i.l20 i.l30 if original==1

foreach a0 of numlist 0,1 { /*index for a0 */

foreach a1 of numlist 0,1 { /*index for a1 */

foreach m of numlist 0,1 { /*index for m */

gen l32_`a0'`a1'_m1_`m' = ind7< 1/(1+exp(-(_b[_cons] + _b[1.l22]*1.l22_`a0'`a1'_m1_`m' + _b[2.l22]*2.l22_`a0'`a1'_m1_`m' /*

*/ + _b[0.l12]*0.l12_`a0'`a1'_m1_`m' + _b[1.l12]*1.l12_`a0'`a1'_m1_`m' + _b[3.l12]*3.l12_`a0'`a1'_m1_`m' /*

*/ + _b[1.a1]*(`a1'==1) + _b[1.m1]*1.G_M1_`m' + _b[0.l11]*0.l11_`a0' + _b[1.l11]*1.l11_`a0' /*

*/ + _b[3.l11]*3.l11_`a0' + _b[1.l21]*1.l21_`a0' + _b[2.l21]*2.l21_`a0' + _b[1.l31]*1.l31_`a0' + /*

*/ _b[1.a0]*(`a0'==1) + _b[1.m0]*1.m0 + _b[1.c1_0]*1.c1_0 + _b[2.c1_0]*2.c1_0 /*

*/ + _b[2.c2_0]*2.c2_0 + _b[3.c2_0]*3.c2_0 + _b[4.c2_0]*4.c2_0 + _b[1.c3_0]*1.c3_0 + _b[1.c4_0]*1.c4_0 + _b[1.c5_0]*1.c5_0 /*

*/ + _b[1.c6_0]*1.c6_0 + _b[1.c7_0]*1.c7_0 + _b[2.c8_0]*2.c8_0 + _b[2.c9_0]*2.c9_0 +_b[3.c9_0]*3.c9_0 + _b[4.c9_0]*4.c9_0 /*

*/ + _b[c10_0]*c10_0 + _b[1.c11_0]*1.c11_0 + _b[2.c11_0]*2.c11_0 + _b[3.c11_0]*3.c11_0 + _b[2.c12_0]*2.c12_0 + _b[3.c12_0]*3.c12_0 + _b[4.c12_0]*4.c12_0 + _b[c13_0]*c13_0 /*

*/ + _b[0.l10]*0.l10 + _b[1.l10]*1.l10 + _b[3.l10]*3.l10 + _b[1.l20]*1.l20 + _b[2.l20]*2.l20 + _b[1.l30]*1.l30)))

}

}

}

*m2: note not required as prediction not subsequently used

logit m2 ib2.l12 i.l22 i.l32 i.a1 i.m1 ib2.l11 i.l21 i.l31 i.a0 i.m0 i.c1_0 i.c2_0 i.c3_0 i.c4_0 i.c5_0 i.c6_0 i.c7_0 i.c8_0 i.c9_0 c10_0 /*

*/ i.c11_0 i.c12_0 c13_0 ib2.l10 i.l20 i.l30 if original==1

foreach a0 of numlist 0,1 { /*index for a0 */

foreach a1 of numlist 0,1 { /*index for a1 */

foreach m of numlist 0,1 { /*index for m */

gen m2_`a0'`a1'_m1_`m' = ind8< 1/(1+exp(-(_b[_cons]+ _b[0.l12]*0.l12_`a0'`a1'_m1_`m' + _b[1.l12]*1.l12_`a0'`a1'_m1_`m' + _b[3.l12]*3.l12_`a0'`a1'_m1_`m' + /*

*/ _b[1.l22]*1.l22_`a0'`a1'_m1_`m' + _b[2.l22]*2.l22_`a0'`a1'_m1_`m' + _b[1.l32]*1.l32_`a0'`a1'_m1_`m' + _b[1.a1]*(`a1'==1) + _b[1.m1]*1.G_M1_`m' + /*

*/ _b[0.l11]*0.l11_`a0' + _b[1.l11]*1.l11_`a0' + _b[3.l11]*3.l11_`a0' + /*

*/ _b[1.l21]*1.l21_`a0' + _b[2.l21]*2.l21_`a0' + _b[1.l31]*1.l31_`a0' + _b[1.a0]*(`a0'==1) + _b[1.m0]*1.m0 + _b[1.c1_0]*1.c1_0 + _b[2.c1_0]*2.c1_0 /*

*/ + _b[2.c2_0]*2.c2_0 + _b[3.c2_0]*3.c2_0 + _b[4.c2_0]*4.c2_0 + _b[1.c3_0]*1.c3_0 + _b[1.c4_0]*1.c4_0 + _b[1.c5_0]*1.c5_0 /*

*/ + _b[1.c6_0]*1.c6_0 + _b[1.c7_0]*1.c7_0 + _b[2.c8_0]*2.c8_0 + _b[2.c9_0]*2.c9_0 +_b[3.c9_0]*3.c9_0 + _b[4.c9_0]*4.c9_0 /*

*/ + _b[c10_0]*c10_0 + _b[1.c11_0]*1.c11_0 + _b[2.c11_0]*2.c11_0 + _b[3.c11_0]*3.c11_0 + _b[2.c12_0]*2.c12_0 + _b[3.c12_0]*3.c12_0 + _b[4.c12_0]*4.c12_0 + _b[c13_0]*c13_0 /*

*/ + _b[0.l10]*0.l10 + _b[1.l10]*1.l10 + _b[3.l10]*3.l10 + _b[1.l20]*1.l20 + _b[2.l20]*2.l20 + _b[1.l30]*1.l30)))

}

}

}

******************************Y***************************************************

logit y i.a2 i.m2 ib2.l12 i.l22 i.l32 i.a1 i.m1 ib2.l11 i.l21 i.l31 i.a0 i.m0 i.c1_0 i.c2_0 i.c3_0 i.c4_0 i.c5_0 i.c6_0 i.c7_0 i.c8_0 i.c9_0 c10_0 /*

*/ i.c11_0 i.c12_0 c13_0 ib2.l10 i.l20 i.l30 if original==1

gen ind9 = runiform()

foreach a0 of numlist 0,1 { /*index for a0 */

foreach a1 of numlist 0,1 { /*index for a1 */

foreach a2 of numlist 0,1 { /*index for a2 */

foreach m1 of numlist 0,1 { /*index for m1 */

foreach m2 of numlist 0,1 { /*index for m2 */

gen y_`a0'`a1'`a2'_m1_`m1'_m2_`m2' = ind9< 1/(1+exp(-(_b[_cons]+ _b[1.a2]*(`a2'==1) + _b[1.m2]*1.G_M2_`m1'`m2' + _b[0.l12]*0.l12_`a0'`a1'_m1_`m1' + _b[1.l12]*1.l12_`a0'`a1'_m1_`m1' + _b[3.l12]*3.l12_`a0'`a1'_m1_`m1' + /*

*/ _b[1.l22]*1.l22_`a0'`a1'_m1_`m1' + _b[2.l22]*2.l22_`a0'`a1'_m1_`m1' + _b[1.l32]*1.l32_`a0'`a1'_m1_`m1' + _b[1.a1]*(`a1'==1) + _b[1.m1]*1.G_M1_`m1' + /*

*/ _b[0.l11]*0.l11_`a0' + _b[1.l11]*1.l11_`a0' + _b[3.l11]*3.l11_`a0' + /*

*/ _b[1.l21]*1.l21_`a0' + _b[2.l21]*2.l21_`a0' + _b[1.l31]*1.l31_`a0' + _b[1.a0]*(`a0'==1) + _b[1.m0]*1.m0 + _b[1.c1_0]*1.c1_0 + _b[2.c1_0]*2.c1_0 /*

*/ + _b[2.c2_0]*2.c2_0 + _b[3.c2_0]*3.c2_0 + _b[4.c2_0]*4.c2_0 + _b[1.c3_0]*1.c3_0 + _b[1.c4_0]*1.c4_0 + _b[1.c5_0]*1.c5_0 /*

*/ + _b[1.c6_0]*1.c6_0 + _b[1.c7_0]*1.c7_0 + _b[2.c8_0]*2.c8_0 + _b[2.c9_0]*2.c9_0 +_b[3.c9_0]*3.c9_0 + _b[4.c9_0]*4.c9_0 /*

*/ + _b[c10_0]*c10_0 + _b[1.c11_0]*1.c11_0 + _b[2.c11_0]*2.c11_0 + _b[3.c11_0]*3.c11_0 + _b[2.c12_0]*2.c12_0 + _b[3.c12_0]*3.c12_0 + _b[4.c12_0]*4.c12_0 + _b[c13_0]*c13_0 /*

*/ + _b[0.l10]*0.l10 + _b[1.l10]*1.l10 + _b[3.l10]*3.l10 + _b[1.l20]*1.l20 + _b[2.l20]*2.l20 + _b[1.l30]*1.l30)))

}

}

}

}

}

***********************

****PART 4

***********************

*baseline: a

sum y_000_m1_0_m2_0

scalar Y000_M1_0_M2_0 = r(mean)

*b

sum y_111_m1_1_m2_1

scalar Y111_M1_1_M2_1 = r(mean)

sum y_111_m1_0_m2_0

scalar Y111_M1_0_M2_0 = r(mean)

scalar rTE_b = Y111_M1_1_M2_1/Y000_M1_0_M2_0

return scalar rTE_b = Y111_M1_1_M2_1/Y000_M1_0_M2_0

scalar rNDE_b = Y111_M1_0_M2_0/Y000_M1_0_M2_0

return scalar rNDE_b = Y111_M1_0_M2_0/Y000_M1_0_M2_0

scalar rNIE_b = Y111_M1_1_M2_1/Y111_M1_0_M2_0

return scalar rNIE_b = Y111_M1_1_M2_1/Y111_M1_0_M2_0

*c

sum y_011_m1_0_m2_1

scalar Y011_M1_0_M2_1 = r(mean)

sum y_011_m1_0_m2_0

scalar Y011_M1_0_M2_0 = r(mean)

scalar rTE_c = Y011_M1_0_M2_1/Y000_M1_0_M2_0

return scalar rTE_c = Y011_M1_0_M2_1/Y000_M1_0_M2_0

scalar rNDE_c = Y011_M1_0_M2_0/Y000_M1_0_M2_0

return scalar rNDE_c = Y011_M1_0_M2_0/Y000_M1_0_M2_0

scalar rNIE_c = Y011_M1_0_M2_1/Y011_M1_0_M2_0

return scalar rNIE_c = Y011_M1_0_M2_1/Y011_M1_0_M2_0

*d

sum y_001_m1_0_m2_0

scalar Y001_M1_0_M2_0 = r(mean)

scalar rTE_d = Y001_M1_0_M2_0/Y000_M1_0_M2_0

return scalar rTE_d = Y001_M1_0_M2_0/Y000_M1_0_M2_0

scalar rNDE_d = Y001_M1_0_M2_0/Y000_M1_0_M2_0

return scalar rNDE_d = Y001_M1_0_M2_0/Y000_M1_0_M2_0

scalar rNIE_d = Y001_M1_0_M2_0/Y001_M1_0_M2_0

return scalar rNIE_d = Y001_M1_0_M2_0/Y001_M1_0_M2_0

restore

end

The program is invoked by the bootstrap command as follows:

bootstrap r(rTE_b) r(rNDE_b) r(rNIE_b) r(rTE_c) r(rNDE_c) r(rNIE_c) r(rTE_d) r(rNDE_d) r(rNIE_d) , seed(1509) reps(500) nowarn : MM_tvar

Example output from the program for 1958-NCDS is reported here:

(running MM_tvar on estimation sample)

Bootstrap replications (500)

----+--- 1 ---+--- 2 ---+--- 3 ---+--- 4 ---+--- 5

.................................................. 50

.................................................. 100

.................................................. 150

.................................................. 200

.................................................. 250

.................................................. 300

.................................................. 350

.................................................. 400

.................................................. 450

.................................................. 500

Bootstrap results Number of obs = 8,674

Replications = 500

command: MM_tvarCorrected

_bs_1: r(rTE_b)

_bs_2: r(rNDE_b)

_bs_3: r(rNIE_b)

_bs_4: r(rTE_c)

_bs_5: r(rNDE_c)

_bs_6: r(rNIE_c)

_bs_7: r(rTE_d)

_bs_8: r(rNDE_d)

_bs_9: r(rNIE_d)

------------------------------------------------------------------------------

| Observed Bootstrap Normal-based

| Coef. Std. Err. z P>|z| [95% Conf. Interval]

-------------+----------------------------------------------------------------

_bs_1 | 1.528104 .2066214 7.40 0.000 1.123134 1.933075

_bs_2 | 1.487058 .200465 7.42 0.000 1.094154 1.879962

_bs_3 | 1.027603 .0103024 99.74 0.000 1.00741 1.047795

_bs_4 | 1.22403 .1327907 9.22 0.000 .9637649 1.484295

_bs_5 | 1.199743 .1300669 9.22 0.000 .9448167 1.45467

_bs_6 | 1.020243 .007298 139.80 0.000 1.005939 1.034547

_bs_7 | 1.141185 .1038865 10.98 0.000 .9375711 1.344799

_bs_8 | 1.141185 .1038865 10.98 0.000 .9375711 1.344799

_bs_9 | 1 . . . . .

------------------------------------------------------------------------------

Where:

_bs_1: r(rTE_b) is the randomised total effect of persistent obesity from 33y

_bs_2: r(rNDE_b) is the randomised natural direct effect of persistent obesity from 33y

_bs_3: r(rNIE_b) is the randomised natural indirect effect (via inactivity) of persistent obesity from 33y

_bs_4: r(rTE_c) is the randomised total effect of incident obesity at 42y

_bs_5: r(rNDE_c) is the randomised natural direct effect of incident obesity at 42y

_bs_6: r(rNIE_c) is the randomised natural indirect effect (via inactivity) of incident obesity at 42y

_bs_7: r(rTE_d) is the randomised total effect of incident obesity at 50y

_bs_8: r(rNDE_d) is the randomised natural direct effect of incident obesity at 50y

_bs_9: r(rNIE_d) is the randomised natural indirect effect (via inactivity) of incident obesity at 50y

Note: for incident obesity at 50y, the randomised total effect is not mediated by inactivity; we assume inactivity precedes obesity (i.e. there is no measure of inactivity between obesity and physical functioning), see supplementary figure 1. Therefore the randomised total effect equals the randomised natural direct effect and the randomised natural indirect effect is 1.

**References**

1. Lin SH, Young J, Logan R, Tchetgen EJT, VanderWeele TJ. Parametric Mediational g-Formula Approach to Mediation Analysis with Time-varying Exposures, Mediators, and Confounders. *Epidemiology* 2017; **28**(2): 266-74.
